# Purine nucleoside phosphorylase enables dual metabolic checkpoints that prevent T cell immunodeficiency and TLR7-associated autoimmunity

**DOI:** 10.1172/JCI160852

**Published:** 2022-08-15

**Authors:** Evan R. Abt, Khalid Rashid, Thuc M. Le, Suwen Li, Hailey R. Lee, Vincent Lok, Luyi Li, Amanda L. Creech, Amanda N. Labora, Hanna K. Mandl, Alex K. Lam, Arthur Cho, Valerie Rezek, Nanping Wu, Gabriel Abril-Rodriguez, Ethan W. Rosser, Steven D. Mittelman, Willy Hugo, Thomas Mehrling, Shanta Bantia, Antoni Ribas, Timothy R. Donahue, Gay M. Crooks, Ting-Ting Wu, Caius G. Radu

**Affiliations:** 1 Department of Molecular and Medical Pharmacology and; 2 Department of Surgery, UCLA, Los Angeles, California, USA.; 3 Department of Nuclear Medicine, Yonsei University College of Medicine, Seoul, South Korea.; 4 Department of Medicine,; 5 Division of Pediatric Endocrinology, UCLA Children’s Discovery and Innovation Institute, and; 6 Division of Dermatology, Department of Medicine, UCLA, Los Angeles, California, USA.; 7 Laevoroc Oncology, Zug, Switzerland.; 8 Parker Institute for Cancer Immunotherapy, San Francisco, California, USA.; 9 Division of Hematology/Oncology, Department of Medicine,; 10 Division of Surgical Oncology, Department of Surgery,; 11 Jonsson Comprehensive Cancer Center,; 12 Division of Pediatric Hematology-Oncology, Department of Pediatrics,; 13 Eli and Edythe Broad Center of Regenerative Medicine and Stem Cell Research, and; 14 Department of Pathology and Laboratory Medicine, UCLA, Los Angeles, California, USA.

**Keywords:** Immunology, Metabolism, Autoimmune diseases, Immunotherapy, T cell development

## Abstract

Purine nucleoside phosphorylase (PNP) enables the breakdown and recycling of guanine nucleosides. PNP insufficiency in humans is paradoxically associated with both immunodeficiency and autoimmunity, but the mechanistic basis for these outcomes is incompletely understood. Here, we identify two immune lineage-dependent consequences of PNP inactivation dictated by distinct gene interactions. During T cell development, PNP inactivation is synthetically lethal with downregulation of the dNTP triphosphohydrolase SAMHD1. This interaction requires deoxycytidine kinase activity and is antagonized by microenvironmental deoxycytidine. In B lymphocytes and macrophages, PNP regulates Toll-like receptor 7 signaling by controlling the levels of its (deoxy)guanosine nucleoside ligands. Overriding this regulatory mechanism promotes germinal center formation in the absence of exogenous antigen and accelerates disease in a mouse model of autoimmunity. This work reveals that one purine metabolism gene protects against immunodeficiency and autoimmunity via independent mechanisms operating in distinct immune lineages and identifies PNP as a potentially novel metabolic immune checkpoint.

## Graphical abstract

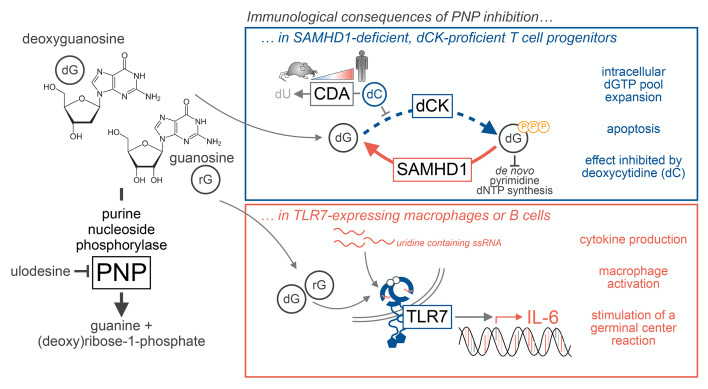

## Introduction

Nucleotide metabolism controls immune cell development and function through diverse mechanisms (1). The purine nucleoside adenosine is sensed by A2_A/B_ receptors, which mediate immunosuppressive effects in various immune cell lineages (2). Although much less studied, guanine nucleosides also appear to possess immunoregulatory properties, as indicated by the phenotypes linked to defects in their catabolism. Purine nucleoside phosphorylase (PNP) is an evolutionarily conserved enzyme that controls the levels of purine nucleosides (guanosine, inosine, and their deoxyribonucleoside analogs) by catalyzing their conversion to a purine nucleobase and (deoxy)ribose-1-phosphate (3). PNP deficiency is a rare autosomal recessive disease in humans associated with highly elevated serum guanosine (rG) and deoxyguanosine (dG) levels, profound T cell immunodeficiency, increased susceptibility to life-threatening recurrent infections, and autoimmunity (4–6). PNP deficiency is one of many immune abnormalities, not all of which involve altered metabolism, associated with immune deficiency and autoimmunity in humans.

It has been suggested that thymic T cell progenitors require PNP activity to prevent the toxic expansion of intracellular deoxyguanosine triphosphate (dGTP) pools derived from excessive dG salvage by nucleoside kinases (7, 8). However, the identity of the nucleoside kinase responsible for the first enzymatic step in the conversion of dG to dGTP in immature T cells lacking PNP activity is unknown, and either cytosolic deoxycytidine kinase (dCK) or mitochondrial dG kinase could enable this process (8). It is also unclear why PNP deficiency selectively affects T and not B lymphocyte development (4), even though PNP is expressed in both lineages (9).

PNP-deficient patients show not only T cell immunodeficiency but also various autoimmune phenotypes, including systemic lupus erythematosus, autoimmune hemolytic anemia, and systemic juvenile idiopathic arthritis with macrophage activation syndrome (5, 6). The hyperactive immune phenotypes of PNP insufficiency appear to be recapitulated in PNP-KO mice, which develop pancytopenia and massive splenomegaly, followed by premature death (8, 10). However, these mice only partially model the manifestations of PNP deficiency in humans and exhibit minor defects in thymopoiesis, while their T cell compartment is largely unaffected (8, 10). It is unclear why PNP inactivation severely affects T cell development in humans but not in mice.

PNP inactivation is paradoxically associated with both immunodeficiency and autoimmunity, but the mechanistic basis for these manifestations is undefined and whether they can be functionally uncoupled remains unknown. To address these gaps in knowledge, we systematically evaluated the immune lineage-dependent consequences of PNP deficiency using a transition state inhibitor that mimics the alterations in guanine nucleoside homeostasis associated with genetic PNP inactivation. Originally developed for the treatment of leukemia, autoimmune disorders, and gout, PNP inhibitors, such as forodesine and ulodesine, rapidly increase serum rG and dG levels by several orders of magnitude in humans and mice and are thereby ideal tools to dissect the functions of PNP in vitro and in vivo (11–13).

Here, we delineated the mechanism underlying the selective effect of PNP insufficiency on T cell development and establish critical roles for dCK and sterile alpha motif and HD domain-containing protein 1 (SAMHD1) in this process. By comparing the effects of PNP inhibition in WT and humanized mice, we uncovered a metabolic basis for the interspecies differences in how PNP insufficiency affects T cell development. Using single-cell transcriptomic and genetic approaches, we revealed a mechanism by which PNP functions to maintain immune tolerance. We showed that PNP inhibition promotes TLR7 activation in B cells and macrophages by increasing the levels of its (deoxy)guanosine nucleoside ligands. We found that chronic PNP inhibition in mice stimulates germinal center formation in secondary lymphoid tissues in the absence of exogenous antigen and accelerates disease in a model of systemic autoimmunity.

Collectively, our findings demonstrate that PNP protects against both immunodeficiency and autoimmunity via hitherto unknown and independent mechanisms operating in distinct immune lineages. They further suggest potentially new approaches to treat PNP insufficiency in humans and identify PNP as a possibly novel metabolic immune checkpoint amenable to pharmacological modulation.

## Results

### dCK and SAMHD1 mediate the selective lethality of PNP deficiency.

**Figure 1:**
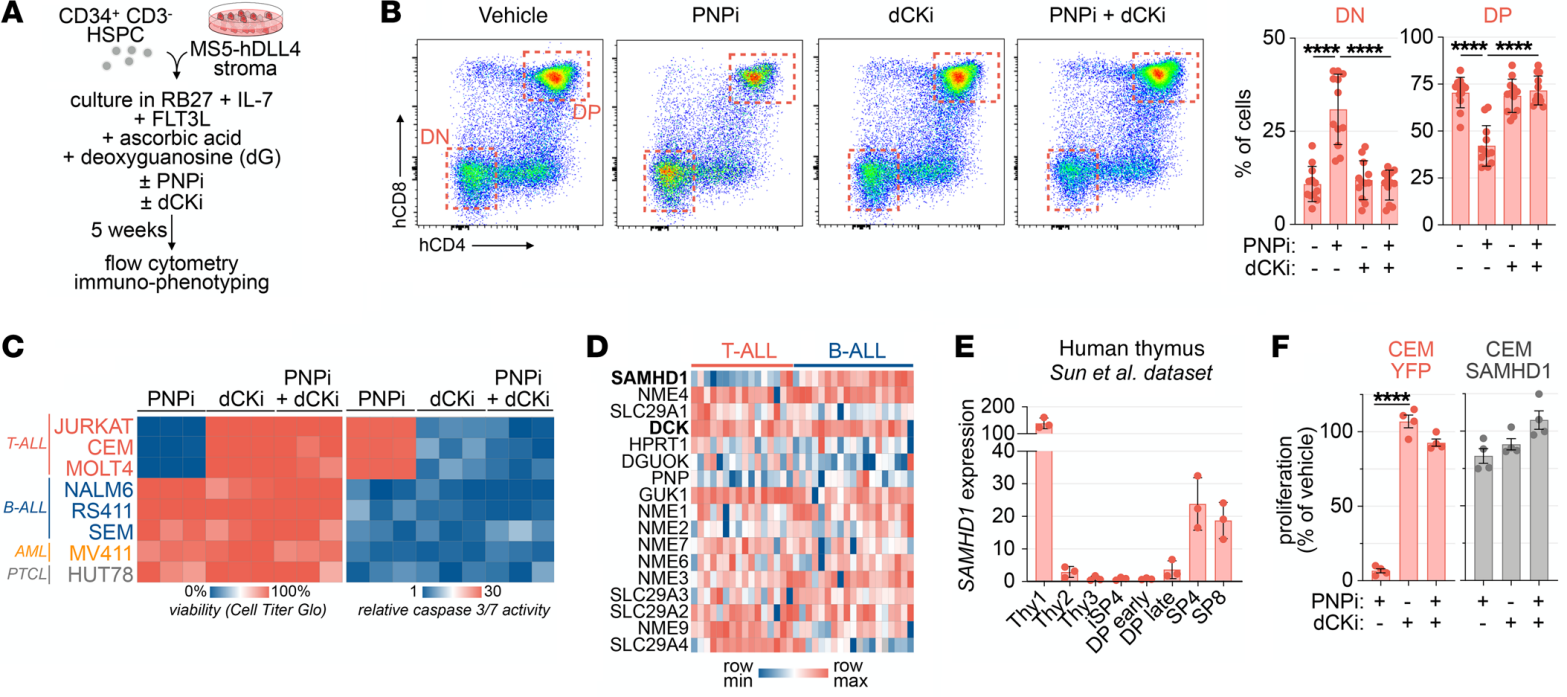
dCK and SAMHD1 mediate PNPi-induced lethality. (**A**) Experimental design for evaluation of PNPi and dCKi in the human artificial thymic organoid (ATO) system. (**B**) Flow cytometry analysis of human ATO T cell development after 5 weeks of continuous treatment with 5 μM deoxyguanosine (dG) with or without 1 μM ulodesine (PNPi) and 1 μM (*R*)-DI-87 (dCKi; mean ± SD; *n =* 12; 1-way ANOVA corrected for multiple comparisons). ATOs were derived from 4 independent donors (*n =* 3/donor). (**C**) Cell Titer Glo and caspase-3/7 glo analysis of cell lines treated with 5 μM dG with or without 1 μM PNPi and 1 μM dCKi for 72 hours (*n =* 3). (**D**) Expression of purine metabolism genes in cancer cell line encyclopedia T- and B-ALL models (Ghandi et al., ref. 19). (**E**) SAMHD1 expression across stages of human thymic T cell development (Sun, et al., ref. 20). (**F**) Cell Titer Glo analysis of CEM-YFP and CEM-SAMHD1 cells treated with 5 μM dG with or without 1 μM PNPi and 1 μM dCKi (mean ± SD; *n =* 4; 1-way ANOVA corrected for multiple comparisons). ****P < 0.0001.

To investigate how PNP inhibition affects human thymopoiesis, we utilized the artificial thymic organoid (ATO) model (14) supplemented with dG and a highly selective and potent PNP inhibitor (PNPi; ulodesine) that rapidly increases serum dG in mice from undetectable levels to 5–20 μM (Supplemental Figure 1A; supplemental material available online with this article; https://doi.org/10.1172/JCI160852DS1), a range similar to that previously observed in PNP-deficient mice and humans (14). In this system, human cord blood–derived hematopoietic stem and progenitor cells (HSPCs) are cultured in defined media as semidry 3D organoids alongside Notch ligand–expressing bone marrow stromal (MS5-hDLL4) cells, which stimulate HSPC differentiation along a developmental trajectory mirroring human thymopoiesis (Figure 1A). Pharmacological PNP inhibition blocks dG catabolism in both stromal and HSPC cell compartments, thereby mimicking dG accumulation in thymi from PNPi-treated mice (Supplemental Figure 1B). To investigate whether dCK plays a role in mediating the effects of PNPi and dG, we used a highly selective and potent dCK inhibitor developed by our group [(*R*)-DI-87; dCKi] (15). After 5 weeks of culture, most T cell precursors in control ATOs progressed from the CD4/CD8 double-negative (DN) to the CD4/CD8 double-positive (DP) stage (Figure 1B). PNP inhibition triggered a developmental block, evidenced by DN cell accumulation, depletion of DP thymocytes, and an approximately 60% decrease in ATO cellularity (Figure 1B and Supplemental Figure 1C). Concomitant dCK inhibition completely rescued the inhibitory effects of PNPi/dG, indicating that this response requires a functional dCK-dependent nucleoside salvage pathway (Figure 1B and Supplemental Figure 1C).

PNP is expressed across immune lineages (9), but PNP deficiency is associated with T cell immunodeficiency (4). To investigate the metabolic mechanisms responsible for this selective effect, we used T cell acute lymphoblastic leukemia (T-ALL) cells, which are derived from immature T cells and have been shown to be highly susceptible to dG-induced toxicity, alongside B-ALL, peripheral T cell lymphoma (PTCL, derived from mature T cells), and acute myeloid leukemia (AML) cells (16, 17). Similar to our findings using primary human thymocytes (Figure 1B), T-ALL cells were sensitive to PNPi/dG in a dCK-dependent manner (Figure 1C). In contrast, B-ALL, AML, and PTCL cells were uniformly resistant (Figure 1C). Furthermore, PNPi/dG did not impair the proliferation of primary murine CD4^+^ or CD8^+^ T lymphocytes activated ex vivo (Supplemental Figure 1D). To determine whether impaired dCK activity underlies innate resistance to PNPi and dG in non-T-ALL lines, we employed an liquid chromatography–tandem mass spectrometry–multiple reaction monitoring (LC-MS/MS-MRM) approach developed by our group (18) to simultaneously evaluate the contributions of dCK-dependent salvage and ribonucleotide reductase–dependent (RNR-dependent) de novo biosynthesis to DNA replication using [^15^N_3_]-labeled deoxycytidine ([^15^N_3_]dC) and [^13^C_6_]glucose, respectively (Supplemental Figure 1E). All tested leukemia and lymphoma cells were capable of utilizing [^15^N_3_]dC for DNA synthesis, indicating that they possess a functional dCK-dependent salvage pathway (Supplemental Figure 1E). Therefore, dCK activity is required but not sufficient for PNPi/dG-mediated lethality.

We reasoned that the activity of other purine metabolism-related genes could dictate sensitivity to PNPi/dG. By examining the expression of such genes across PNPi/dG-sensitive T-ALL versus resistant B-ALL models, we observed that the deoxyribonucleotide triphosphohydrolase SAMHD1 was expressed at low levels in the majority of cancer cell line encyclopedia–annotated (CCLE-annotated) T-ALL lines relative to B-ALL lines (Figure 1D and Supplemental Figure 1F) (19). Analysis of SAMHD1 expression in T-ALL cells versus B-ALL cells included in PRoXe, a public repository of patient-derived leukemia cells (https://www.proxe.org/), revealed a similar pattern, with most T-ALL cells lacking detectable SAMHD1 expression, and the majority of B-ALL cells expressing SAMHD1; dCK was expressed at similar levels in T-ALL cells and B-ALL cells (Supplemental Figure 1G). Moreover, DN murine and human thymocytes, which are sensitive to PNPi/dG, express much lower levels of SAMHD1 than PNPi/dG-resistant mature CD4^+^ and CD8^+^ single-positive T cells (Figure 1E and Supplemental Figure 1H) (20). In contrast, SAMHD1 expression was detectable throughout B cell development (Supplemental Figure 1H), which is largely unaffected by PNP deficiency (4, 21).

To confirm that SAMHD1 protects against PNPi/dG mediated toxicity, we restored SAMHD1 expression in CCRF-CEM (CEM) T-ALL cells (Supplemental Figure 1I) (22). In SAMHD1-deficient CEM-YFP cells, PNPi/dG abolished proliferation and induced markers of replication stress (CHEK1-S345 phosphorylation), DNA damage (H2A.X-S139 phosphorylation), and apoptosis (annexin V), while SAMHD1-expressing isogenic cells were completely resistant (Figure 1F and Supplemental Figure 1, J and K).

**Figure 2:**
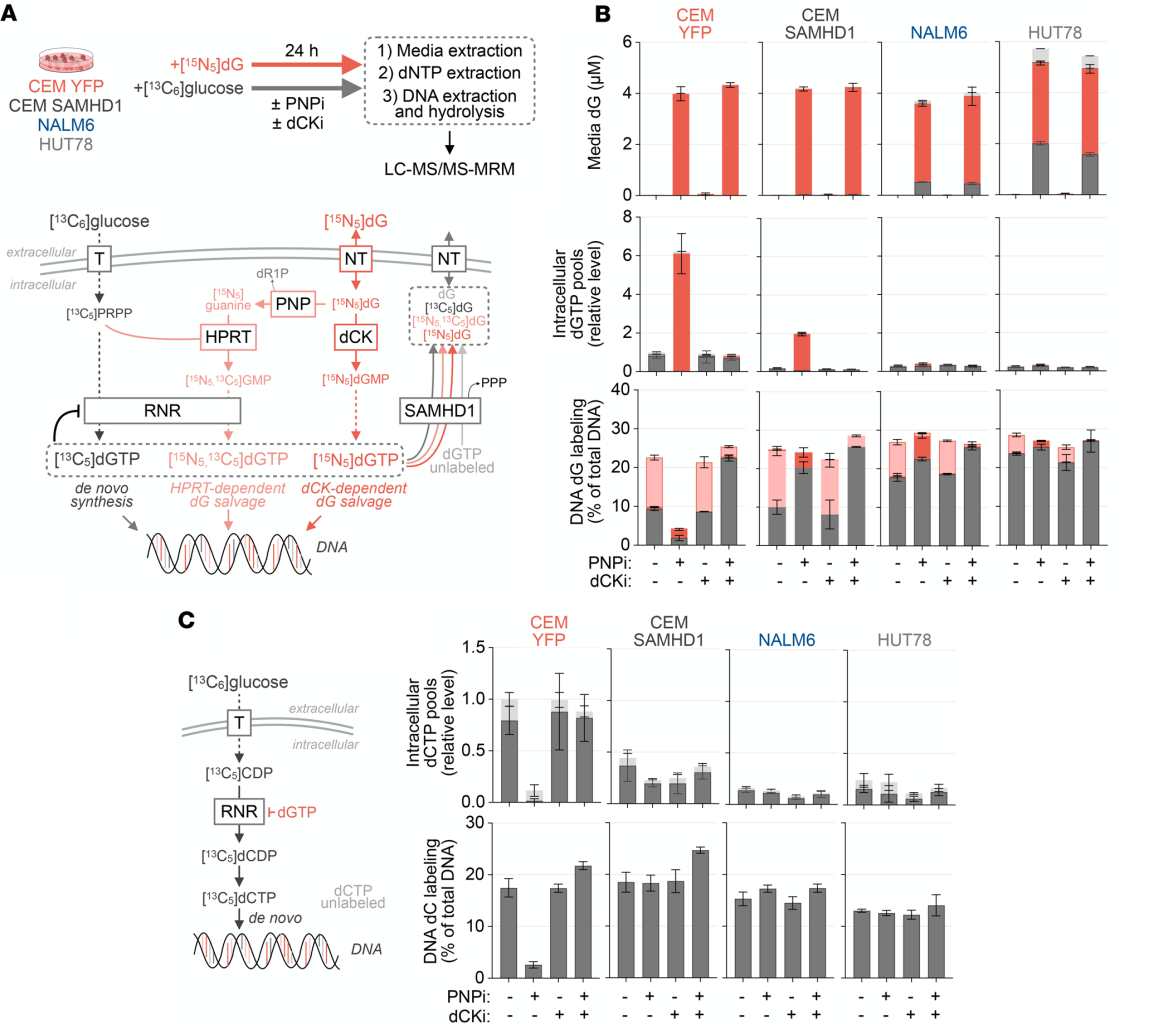
PNPi-induced metabolic alterations are modulated by dCK and SAMHD1. (**A**) Schematic of stable isotope-labeled metabolite-tracing approach to track the utilization of glucose and dG for dGTP pool synthesis and DNA replication. (**B**) LC-MS/MS-MRM analyses of cellular dGTP pools, media dG, and DNA deoxyguanosine composition in cells treated for 24 hours with or without 1 μM PNPi and 1 μM dCKi in the presence of 1 g/L [^13^C_6_]glucose and 5 μM [^15^N_5_]dG. Bar colors indicate metabolite stable isotope composition (mean ± SD; *n =* 4). (**C**) LC-MS/MS-MRM analyses of intracellular dCTP pool and DNA deoxycytidine composition from experiment in **A** (mean ± SD; *n =* 4). T, glucose transporter; NT, nucleoside transporter; dR1P, deoxyribose-1- phosphate.

SAMHD1 catalyzes the phosphohydrolysis of deoxyribonucleoside triphosphates (dNTPs) to nucleosides and is a critical regulator of intracellular dNTP abundance (23). To evaluate the effects of PNPi/dG and dCKi on dNTP pools and DNA replication on SAMHD1-proficient and -deficient cells, we performed LC-MS/MS-MRM tracing of stable isotope-labeled glucose ([^13^C_6_]glucose)- and dG ([^15^N_5_]dG)-derived metabolites. As depicted schematically in Figure 2A, dGTP isotopologue fractional enrichment delineates the biosynthetic route by which it was derived, either by de novo synthesis from glucose via RNR (identified by [^13^C_5_]dGTP) or by salvage of intact dG via dCK (identified by [^15^N_5_]dGTP). This approach also traces the contribution of guanine nucleobases liberated from dG by PNP to dGTP pools via hypoxanthine phosphoribosyltransferase–mediated (HPRT-mediated) fusion with glucose-derived phosphoribosyl pyrophosphate (identified by [^15^N_5_,^13^C_5_]dGTP). In SAMHD1-proficient cells, PNPi prevented HPRT-dependent guanine nucleobase salvage, but RNR-dependent de novo synthesis fully compensated for this defect and sustained DNA synthesis (Figure 2B). In SAMHD1-deficient cells, PNPi massively expanded the dGTP pool derived from dCK-mediated salvage of intact [^15^N_5_]dG. This effect was blocked by dCKi, substantially reduced in CEM-SAMHD1 cells, and absent in NALM6 (B-ALL) and HUT78 (PTCL) cells, which endogenously express SAMHD1. In PNPi-treated SAMHD1-deficient cells, dGTP pool expansion was associated with inhibition of DNA replication. PNPi impaired pyrimidine dNTP synthesis in these cells, as evidenced by a restriction of dCTP abundance, decreased de novo dCTP production, and reduced dCTP utilization for DNA replication (Figure 2C). Mechanistically, the restriction of dCTP pools by PNPi/dG in SAMHD1-deficient cells likely reflects a dGTP-mediated allosteric switch in RNR substrate specificity that favors ADP reduction at the expense of CDP reduction (24).

Next, we determined whether the synthetic lethal interaction between PNP and SAMHD1 inactivation extends to solid tumors. HCC827 lung adenocarcinoma cells exhibited the lowest SAMHD1 expression among CCLE-annotated solid tumor cells (Supplemental Figure 2, A and B) and were highly susceptible to PNPi/dG in a dCK-dependent manner (Supplemental Figure 2C). Restoring SAMHD1 expression in HCC827 cells rendered them completely resistant to PNPi/dG-mediated toxicity (Supplemental Figure 2, B and C). Conversely, knocking out SAMHD1 in SUIT2 pancreas cancer cells rendered them hypersensitive to the dCK-dependent lethality of PNPi/dG (Supplemental Figure 2, B and C). As SAMHD1 expression was undetectable in a subset of CCLE melanoma cell lines (Supplemental Figure 2A), we extended our analysis to a panel of melanoma cell lines derived at UCLA. We identified 2 lines (M230 and M418) in which SAMHD1 expression was undetectable both at baseline (Supplemental Figure 2E) and after type I IFN (IFN-β) treatment; in contrast, SAMHD1 was expressed at baseline in M297, M257, and M296 lines and was further increased by IFN-β exposure (Supplemental Figure 2F), consistent with SAMHD1 being an IFN-stimulated gene (25). PNPi/dG elicited dCK-dependent proliferation inhibition in SAMHD1-deficient M230 and M418 cells, while having no effects against SAMHD1-proficient M297 cells (Supplemental Figure 2G). In summary, these findings identify critical roles for dCK and SAMHD1 as mediators for the cellular lethality of PNP inactivation in both lymphocyte and nonlymphocyte models.

### Metabolic basis for interspecies differences in the effect of PNP deficiency on T cell development.

**Figure 3:**
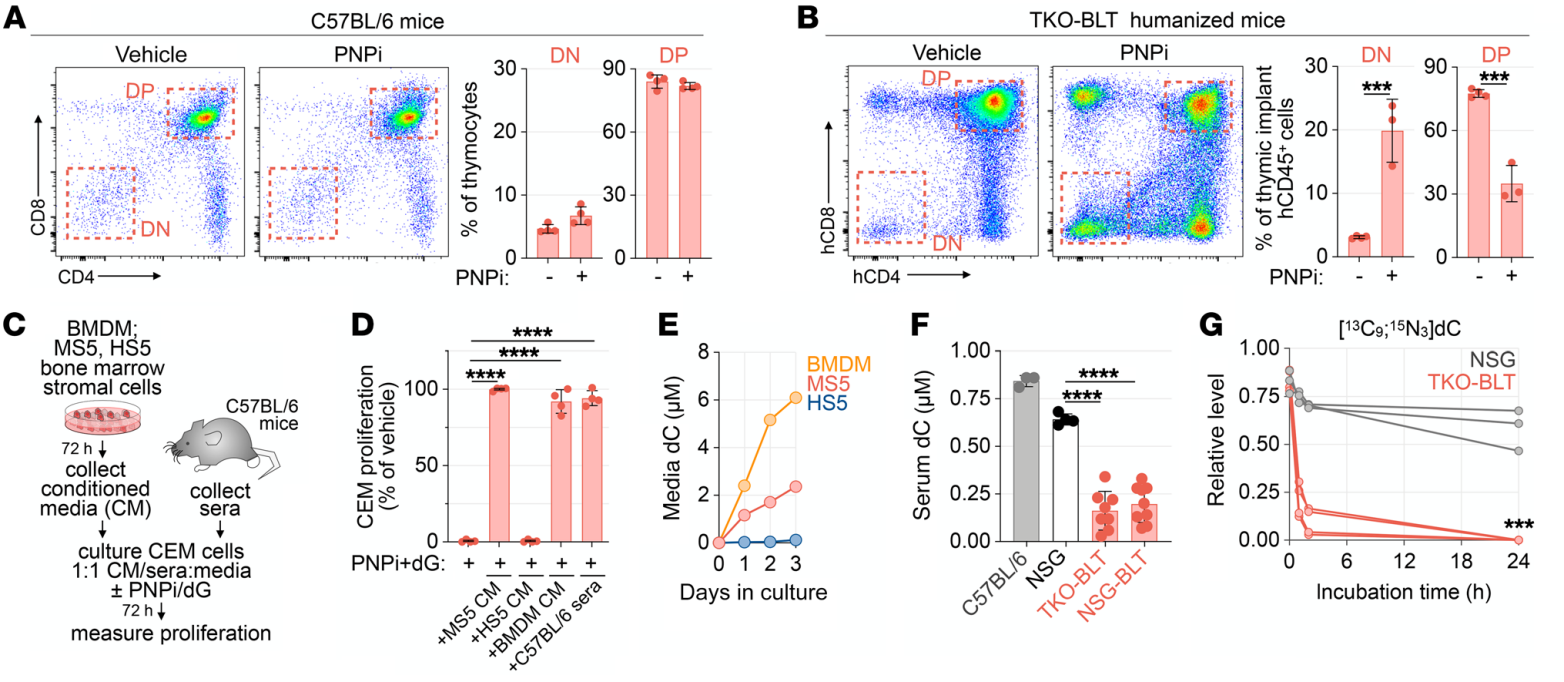
dC is an environmental factor that dictates PNPi lethality. (**A**) Cellular composition of thymi from C57BL/6 mice treated with or without PNPi (100 mg/kg; p.o.; q.d.) for 14 days (mean ± SD; *n =* 4). (**B**) Human thymic implant cellular composition in humanized TKO-BLT mice treated with or without PNPi (100 mg/kg; p.o.; q.d.) for 13 days. Plot indicates the percentage of human CD45^+^ (hCD45^+^) cells (*n =* 4 vehicle; *n =* 3 PNPi; mean ± SD; 2-tailed Mann-Whitney test). (**C**) Schematic of bone marrow–derived macrophage (BMDM) and stroma cell–conditioned media (CM)/serum rescue experiments. (**D**) Cell Titer Glo analysis of CEM cells treated with 1 μM PNPi and 5 μM dG in 1:1 base media to CM/sera (mean ± SD; *n =* 4; 1-way ANOVA corrected for multiple comparisons). (**E**) LC-MS/MS-MRM analysis of dC in CM (mean ± SD; *n =* 3). (**F**) LC-MS/MS-MRM analysis of serum dC levels in C57BL/6, NCG (*n =* 3), and BLT humanized mice (*n =* 8 group; mean ± SD; 2-tailed Mann-Whitney test). (**G**) LC-MS/MS-MRM analysis of ex vivo [^13^C_9_;^15^N_3_]dC catabolism in NSG and TKO BLT sera (*n =* 3 NSG; *n =* 4 TKO-BLT; 2-tailed Mann-Whitney test). ***P < 0.001; ****P < 0.0001.

PNP-KO mice manifest minor alterations in T cell development and fail to recapitulate the severe T cell immunodeficiency observed in humans with inactivating mutations in PNP (8, 26). Consistent with prior studies in PNP-KO mice, but in contrast to our data in the human ATO system (Figure 1B), we did not observe alterations in thymocyte developmental stage distribution in C57BL/6 mice treated with PNPi for 2 weeks (Figure 3A). To investigate a potential metabolic basis for this interspecies difference, we used 2 orthogonal approaches. First, we examined the effects of PNP inhibition on thymopoiesis in a bone marrow, liver, thymus–humanized (BLT-humanized) mouse model generated by engrafting human fetal liver and thymic fragments under the renal capsule and intravenously delivering hematopoietic stem cells into immunodeficient NOD/SCID IL2Rg^null^ (NSG) or triple-KO (TKO; lacking RAG2, CD47, and X-linked IL2RG) mice (27). As shown in Figure 3B, PNP inhibition significantly impaired human T cell development in the mice, as evidenced by a CD4/CD8 DN-stage developmental block. which resembles the phenotype observed in the ATO system (Figure 1B). Second, we evaluated the effects of (a) conditioned media (CM) derived from murine (MS5) or human (HS5) bone marrow mesenchymal stromal cell lines, (b) CM from murine bone marrow–derived macrophages (BMDMs), and (c) C57BL/6 mouse sera on PNPi/dG response in SAMHD1-deficient CEM cells (Figure 3C). Strikingly, MS5 and BMDM-derived CM as well as mouse sera completely rescued PNPi/dG-induced lethality, while HS5-derived CM lacked such protective effects (Figure 3D).

Together, these data suggest the existence of a soluble factor that renders SAMHD1-deficient thymocytes and T-ALL cells resistant to PNPi/dG. This factor is present in sera from C57BL/6 mice and CM from murine bone marrow stromal cells and BMDMs but not in sera from humanized mice or CM from human bone marrow stromal cells. We focused on nucleosides as potential resistance factors, as previous work by us and others has shown that stromal cells release these metabolites (25, 28). LC-MS/MS-MRM analyses revealed that resistance-conferring MS5 CM contained substantially more nucleosides uridine, cytidine, and deoxycytidine (dC) than the HS5 CM (Figure 3E and Supplemental Figure 3A). dC emerged as the most plausible resistance factor, given that both dC and dG are salvaged via dCK. dC, however, is the preferred dCK substrate, as indicated by the specificity constant (K_cat_/K_M_) values 23.1 × 10^3^ and 2.9 × 10^3^ for dC and dG, respectively (29). dC levels were also elevated in the resistance-conferring CM from BMDMs (Figure 3E). Consistent with a role for dC in mediating PNPi response in vivo, serum dC levels in BLT mice were significantly lower than in naive NSG mice or C57BL/6 mice and approached levels measured in humans and nonhuman primates (Figure 3F) (30). Moreover, humanized mouse sera exhibited significantly increased dC degradation capacity compared with naive NSG sera (Figure 3G), suggesting an important role for catabolism in regulating dC serum levels.

**Figure 4:**
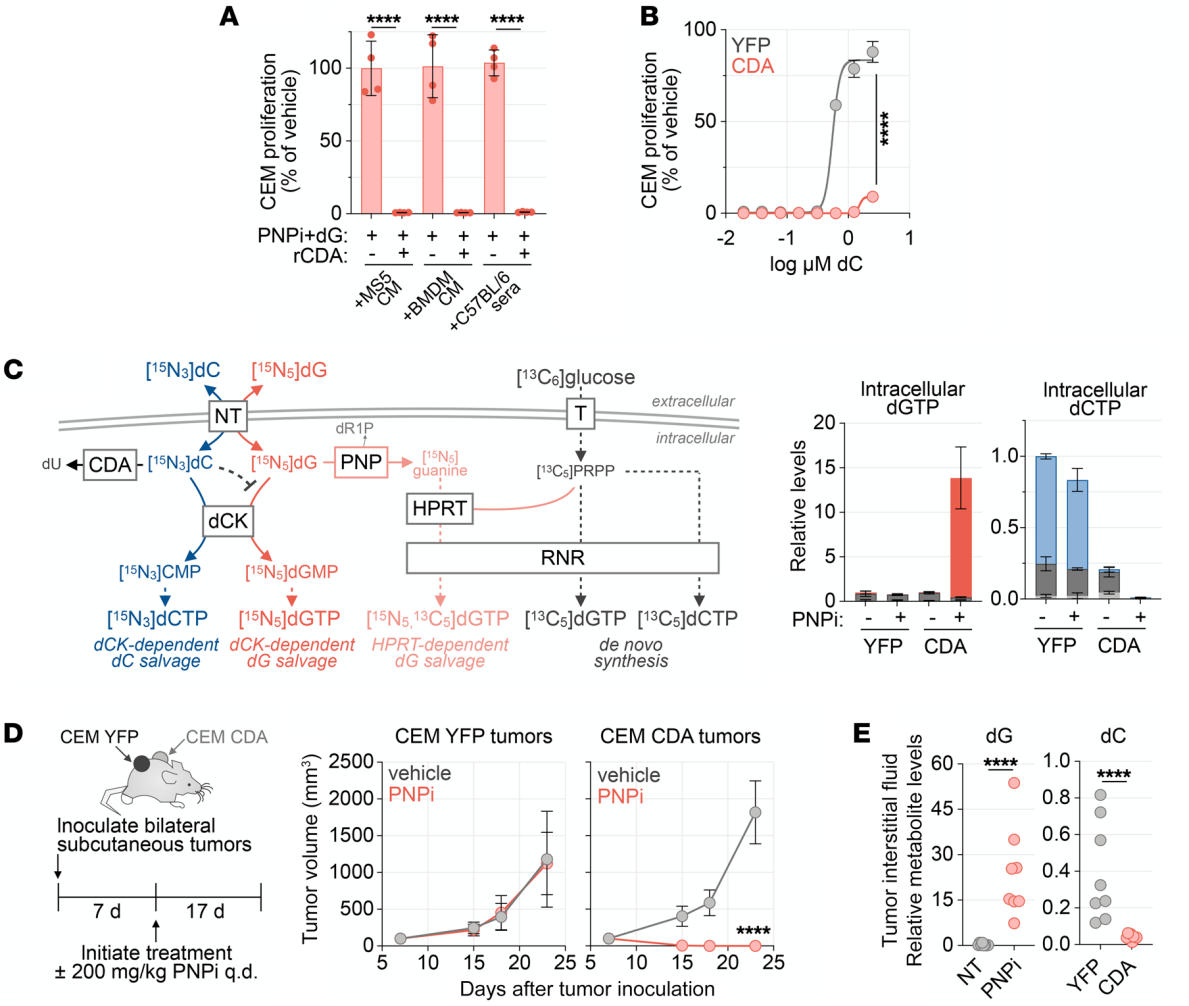
CDA prevents dC-mediated PNPi resistance. (**A**) Cell Titer Glo analysis of CEM cells treated with 1 μM ulodesine (PNPi)/5 μM dG with or without 10 ng/ml recombinant cytidine deaminase (rCDA) in 1:1 base media to conditioned media (CM; *n =* 4). (**B**) Cell Titer Glo analysis of CEM-YFP and CEM-CDA cells treated with 5 μM dG and 1 μM PNPi with or without dC for 72 hours (*n =* 4; mean ± SD). (**C**) Stable isotope-labeled metabolite tracing and LC-MS/MS-MRM analysis of intracellular dNTP in CEM-YFP control or CEM-CDA cells treated for 24 hours with or without 1 μM PNPi in the presence of 1 g/L [^13^C_6_]glucose, 5 μM [^15^N_3_]dC, and 5 μM [^15^N_5_]dG. Bar color indicates metabolite isotopic composition and biosynthetic route by which it was formed (mean ± SD; *n =* 4). (**D**) μCT tumor volume analysis of PNPi response in NCG mice bearing bilateral CEM-YFP and CEM-CDA tumors treated with vehicle or PNPi (200 mg/kg, p.o. q.d.; mean ± SD; *n =* 8; 2-tailed Mann-Whitney test). (**E**) LC-MS/MS-MRM analysis of tumor interstitial fluid composition from the endpoint of experiment in **D** (*n =* 8; unpaired *t* test). CEM-YFP tumors were used for dG comparison, and vehicle-treated tumors were used for dC comparison. ****P < 0.0001.

To confirm a role for dC catabolism in modulating responses to PNPi/dG, we used the dC-catabolizing enzyme cytidine deaminase (CDA). The addition of recombinant CDA abrogated the resistance to PNPi/dG conferred by dC, MS5/BMDM-derived CM, and C57BL/6 mouse serum (Figure 4A and Supplemental Figure 3B). Similar effects were observed using SAMHD1-deficient CEM and HCC827 cells engineered to stably express CDA (Supplemental Figure 3C). dC supplementation abrogated PNPi/dG lethality in parental cells but ectopic CDA expression prevented this rescue (Figure 4B and Supplemental Figure 3D).

We performed [^13^C_6_]glucose, [^15^N_5_]dG, and [^15^N_3_]dC tracing and LC-MS/MS analysis in CEM-YFP or CEM-CDA cells to gain additional mechanistic insight into the interplay between dC and CDA in modulating PNPi/dG-mediated toxicity (Figure 4C and Supplemental Figure 3E). Ectopic CDA expression resulted in the depletion of [^15^N_3_]dC from the media (Supplemental Figure 3F). PNPi expanded the dGTP pool derived from [^15^N_5_]dG salvage and depleted intracellular dCTP only in CEM-CDA cells and had no effect in the parental cells in the presence of dC (Figure 4C). This indicated that dC blocks the entry of dG into dGTP pools via dCK and thereby acts as an endogenous inhibitor of PNPi/dG-mediated lethality.

To investigate whether increasing CDA levels in vivo sensitizes SAMHD1-deficient cells to PNPi, we established CEM-YFP control and CEM-CDA bilateral subcutaneous tumors in NCG mice and initiated PNPi treatment once tumor volumes reached 100 mm^3^ (Figure 4D). PNPi treatment eradicated CEM-CDA tumors, while CEM-YFP tumors grew unchecked (Supplemental Figure 3G). LC-MS/MS-MRM analyses of tumor interstitial fluid dG and dC levels showed that PNPi increased dG levels while CDA expression significantly reduced dC levels (Figure 4E). Collectively, these findings indicate that elevated dC levels in mice relative to humans or humanized mice engender resistance to the synthetic lethality between PNP and SAMHD1 and suggest that humanized mice are a suitable model for the metabolic alterations induced by PNP deficiency during T cell development.

### PNP inhibition in mice induces immune activation phenotypes.

**Figure 5:**
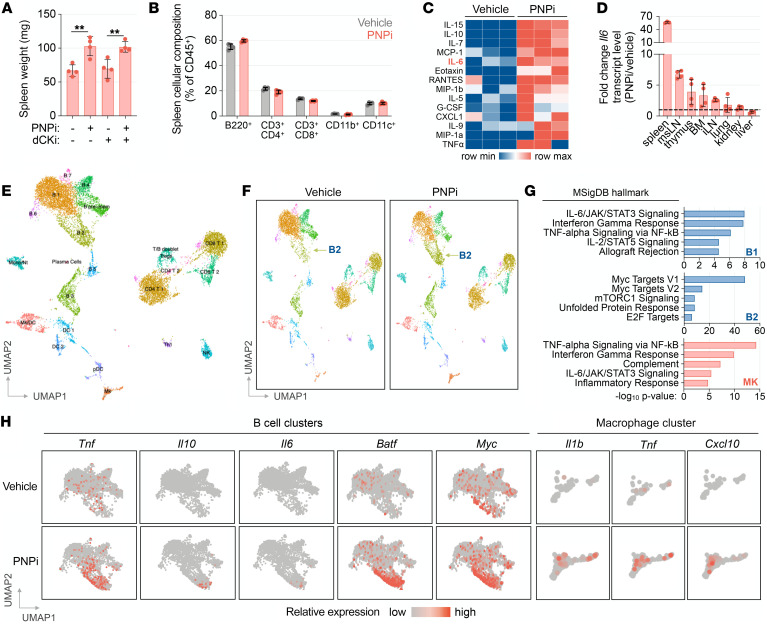
PNPi elevates cytokine levels and modifies the transcriptional phenotype of splenocytes. (**A**) Analysis of C57BL/6 mice treated with or without 100 mg/kg ulodesine (PNPi; p.o.; q.d.) and 75 mg/kg (*R*)-DI-87 (dCKi; p.o.; b.i.d.) for 13 days (*n =* 4; Kruskal-Wallis test with Dunn’s multiple comparisons correction). (**B**) Flow cytometry analysis of spleen cellular composition from experiment in **A**. (**C**) Luminex serum cytokine profile of C57BL/6 mice 6 hours after treatment with or without PNPi (100 mg/kg; p.o.). (**D**) RT-PCR analysis of tissue cytokine transcript levels from C57BL/6 mice 6 hours after treatment with or without PNPi (100 mg/kg; *n =* 4). (**E**) UMAP projection of splenocytes (6701 from the vehicle-treated group and 6412 from the PNPi-treated group). scRNA-Seq analysis was performed on splenocytes isolated from C57BL/6 mice 6 hours after treatment with or without PNPi (100 mg/kg; p.o.; splenocytes from 3 mice/group were pooled before sequencing). (**F**) UMAP projection and clustering of vehicle and PNPi groups. (**G**) Ontology analysis of significantly altered transcripts in select B cell (B1 and B2) and macrophage (MK) clusters. (**H**) Feature plot analysis of PNPi-induced expression alterations in B cell and macrophage clusters. **P < 0.01.

In addition to T cell immunodeficiency, loss of PNP function associates with various autoimmune and inflammatory phenotypes in humans (5). Moreover, PNP-KO mice exhibit phenotypes indicative of hyperimmune activation, such as splenomegaly (8, 10). Consistently, pharmacological PNP inhibition in C57BL/6 mice for 2 weeks induced a significant increase in spleen size (Figure 5A). This effect occurred independently of dCK activity and was not accompanied by alterations in spleen cellular composition (Figure 5B).

To elucidate the mechanism responsible for this phenotype, we first determined whether acute PNP inhibition in C57BL/6 mice alters serum levels of cytokines associated with immune activation. A single administration of PNPi was sufficient to induce multiple cytokines and chemokines (Figure 5C). In particular, we noted a robust increase in IL-6, an inflammatory cytokine linked to autoimmunity and inflammation in mice and humans (31, 32). To identify the source(s) of IL-6 in PNPi-treated mice, we measured *Il6* transcript levels across multiple tissues and found that the spleen exhibited the greatest fold increase in *Il6* transcripts, followed by the lymph nodes and the thymus (Figure 5D).

To further investigate the effects of acute PNP inhibition, we performed single-cell RNA-Seq (scRNA-Seq) analysis of splenocytes isolated from vehicle- and PNPi-treated mice. Integrated uniform manifold approximation and projection (UMAP) of splenocytes from control and treated mice identified 23 unique clusters encompassing all major immune cell lineages (Figure 5E). The most profound transcriptional alterations induced by PNP inhibition were observed within an expanded B2 cluster, which corresponds to activated CD19^+^ B cells, and within the CSF1R^+^ macrophage cluster (Figure 5F and Supplemental Figure 4A). Ontological analysis of genes significantly altered by PNPi revealed enrichment of pattern recognition receptor (PRR) signaling–driven NF-κB–mediated cytokine responses and IFN regulatory factors/IFN response signatures across CD19^+^ B cell and CSF1R^+^ macrophage clusters (Figure 5G and Supplemental Figure 4B). PNPi additionally stimulated Myc and E2f transcription factor–regulated responses associated with proliferation in cluster B2 cells (Figure 5G). Furthermore, PNP inhibition stimulated NF-κB–regulated cytokine transcripts, including *Tnf*, *Il10*, and *Il6* in B cells, alongside *Tnf*, *Il1b*, and IFN-response biomarker *Cxcl10* in macrophages (Figure 5H). These findings indicate that, in addition to its positive role in T cell development, PNP functions in the periphery as a negative regulator of immune activation initiated by PRR signaling. These immune functions of PNP are likely mediated by different mechanisms involving distinct gene interactions.

### PNP controls TLR7 at the level of guanosine ligand availability.

**Figure 6:**
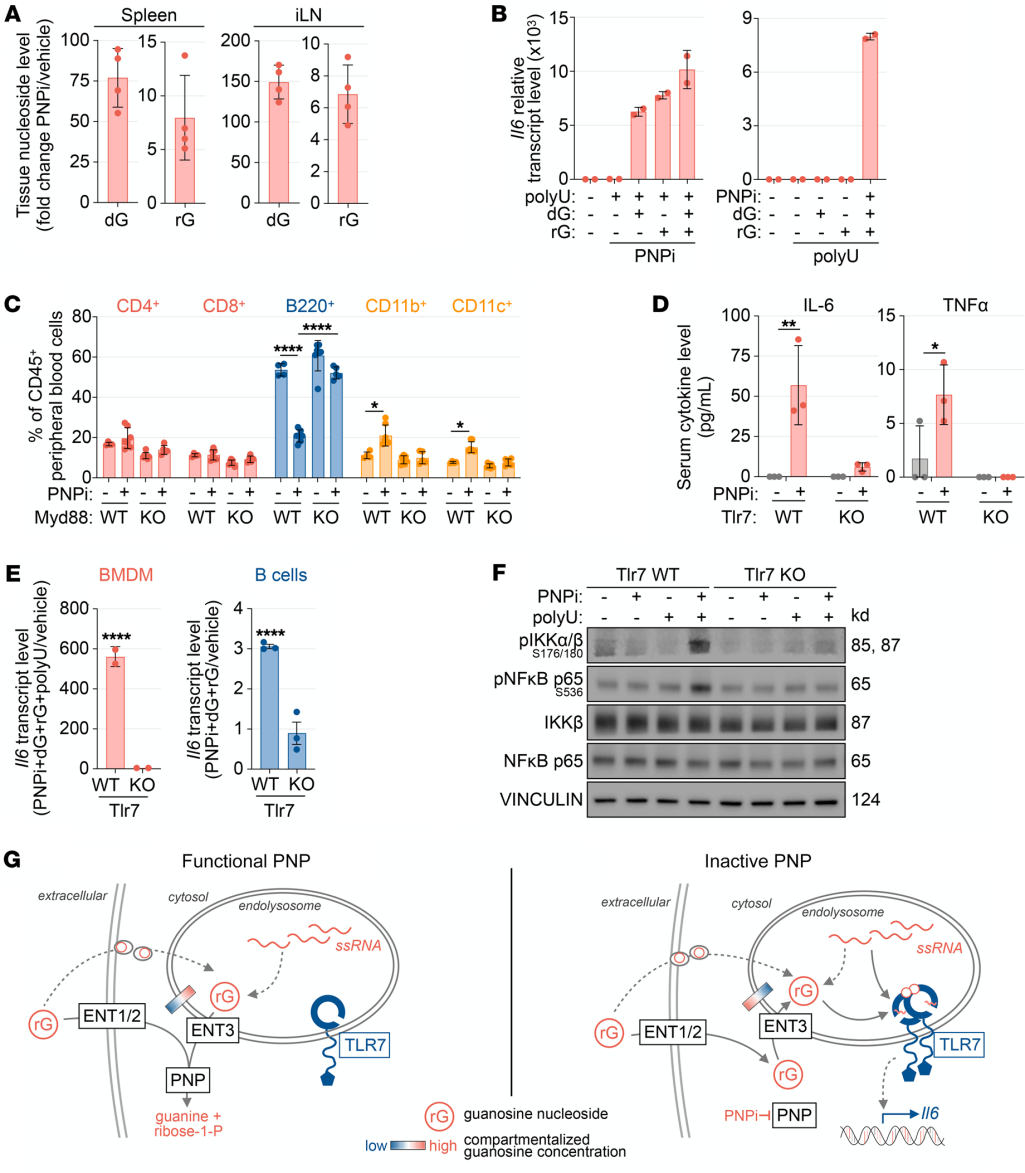
PNP is a negative regulator of TLR7. (**A**) LC-MS/MS-MRM analysis of spleen and inguinal lymph node (iLN) metabolite composition in C57BL/6 mice 24 hours after treatment with or without 100 mg/kg ulodesine (PNPi; mean ± SD; *n =* 4; unpaired *t* test). (**B**) RT-PCR analysis of bone marrow–derived macrophages (BMDMs) treated with or without 1 μM PNPi, 5 μg polyU ssRNA (complexed with DOTAP), 5 μM guanosine (rG), and 5 μM deoxyguanosine (dG) for 4 hours (mean ± SD; *n =* 2). (**C**) Flow cytometry analysis of peripheral blood in C57BL/6 WT and Myd88-KO mice treated with or without PNPi (100 mg/kg; p.o.; q.d.) for 7 days (mean ± SD; *n =* 4–7; 1-way ANOVA corrected for multiple comparisons). (**D**) Serum cytokine profiles of C57BL/6 WT or Tlr7-KO mice 6 hours after treatment with or without PNPi (100 mg/kg p.o.; *n =* 3; 1-way ANOVA corrected for multiple comparisons). (**E**) RT-PCR analysis of BMDMs and CD43^–^ splenic B cells derived from WT or Tlr7-KO mice stimulated with or without 1 μM PNPi and 5 μM dG/rG for 4 hours (*n =* 3; mean ± SD; 1-way ANOVA corrected for multiple comparisons). (**F**) WT or Tlr7-KO BMDMs treated with or without PNPi (+ 5 μM dG + 5 μM rG) and 5 μg polyU complexed with DOTAP for 1 hour. (**G**) Model for the regulation of endolysosomal guanosine nucleoside levels by PNP. *P < 0.05; **P < 0.01; ****P < 0.0001.

Recent studies of TLR7 and TLR8 activation mechanisms provide potential clues about how PNP may function as a negative regulator of PRR activation (33, 34). TLR7 activation requires binding of single-stranded RNA (ssRNA) containing a nonterminal uridine residue to “site 2” followed by binding of a guanine nucleoside to “site 1” (33). Dual ssRNA/guanine nucleoside binding initiates TLR7 dimerization, activation of downstream signaling, and transcriptional initiation of cytokine responses. We reasoned that PNP inactivation could initiate TLR7 signaling by increasing the levels of “site 1” ligands. Consistent with this model, acute inhibition of PNP in mice substantially increased guanine nucleoside levels in the spleen and lymph nodes, which correlated with the observed induction of IL-6 (Figure 6A).

To investigate a role for PNP as a negative regulator of TLR7, we utilized TLR7-expressing BMDMs stimulated with combinations of TLR7 site 1 (dG and rG) and site 2 (21-mer poly-uridine RNA complexed with DOTAP [polyU]) ligands with or without PNPi. Both dG and rG induced *Il6* expression in PNPi- and polyU-treated BMDMs (Figure 6B). PNP inhibition was required for the ability of guanosine nucleosides to induce IL-6 expression in polyU-treated BMDMs (Figure 6B). In BMDMs, PNPi and guanosine nucleosides were also essential for the ability of various polyU concentrations to induce *Il6* expression (Supplemental Figure 5A) and phosphorylation of downstream TLR7 signaling mediators, IKKβ, IκBα, and NF-κB (Supplemental Figure 5B). Inhibition of TAK1, IRAK4, or IKKβ, the kinases responsible for propagating TLR7 signaling, restricted *Il6* induction upon PNPi+dG/rG+ssRNA treatment (Supplemental Figure 5C). While polyU induced *Ifnb1* in expression in BMDMs in a dose-dependent manner, PNPi and guanine nucleosides significantly increased this response at all examined polyU concentrations (Supplemental Figure 5D). PNP inhibition also modulated ssRNA recognition in other TLR7-expressing immune cells. In FLT3-differentiated bone marrow–derived dendritic cells, PNPi+dG/rG significantly potentiated polyU-induced *Il6* expression (Supplemental Figure 5E). In primary murine B cells, PNPi+dG/rG induced *Il6* expression independently of polyU (Supplemental Figure 5E), indicating that these cells contain sufficient amounts of endogenous ssRNA to initiate TLR7 signaling in the presence of elevated levels of site 1 guanosine ligands.

Next, we used genetic approaches to unequivocally demonstrate that PNP inhibition triggers TLR7 activation. Chronic TLR7 signaling in mice is associated with reductions of B cell numbers in peripheral blood and expansion of myeloid cell populations (35). Consistently, PNPi treatment in mice restricted circulating B220^+^ B cells while expanding CD11b^+^ and CD11c^+^ myeloid populations (Figure 6C). Genetic inactivation of MyD88, an essential TLR7 signaling adaptor, prevented these alterations. Additionally, we observed that (a) increases in serum IL-6 and TNF-α by PNPi were abrogated in Tlr7-KO mice (Figure 6D), (b) PNPi+dG/rG did not induce *Il6* expression in polyU-transfected Tlr7-KO BMDMs or B cells (Figure 6E), and (c) PNPi+dG/rG induced phosphorylation of downstream TLR7 effectors IKKα/β and NF-κB p65 alongside polyU only in TLR7-expressing BMDMs (Figure 6F).

Collectively, these findings suggest a model in which PNP regulates TLR7 activation by restricting the availability of site 1 guanosine ligands in endolysosomes (Figure 6G). PNP inactivation stabilizes guanosine nucleosides, thereby facilitating their transit to endolysosomes.

### Unique transcriptional responses to PNPi-stabilized guanosine nucleosides and imidazoquinolines.

**Figure 7:**
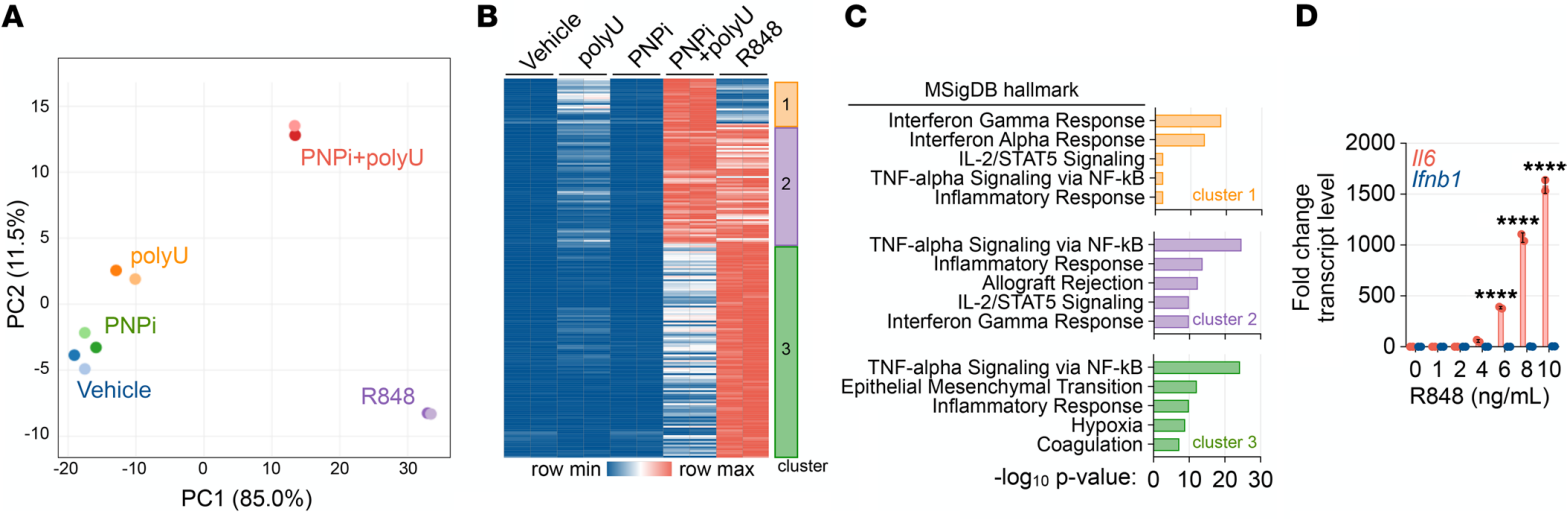
Distinct responses triggered by endogenous or synthetic TLR7 ligands. (**A**) Principal component analysis (PCA) of transcriptome alterations in BMDMs treated with or without PNPi (1 μM ulodesine, 5 μM dG, and 5 μM rG), 5 μg polyU complexed with DOTAP, and 1 ng/mL R848 for 4 hours measured using RNA-Seq (*n =* 2). (**B**) K-means clustering of significantly upregulated transcripts (>10 fold change, <0.01% FDR in 1 pairwise comparison) from experiment in **A**. (**C**) MSigDB hallmark ontology enrichment analysis. (**D**) RT-PCR analysis of BMDMs treated with or without indicated doses of R848 for 4 hours (mean ± SD; *n =* 2; 1-way ANOVA corrected for multiple comparisons). ****P < 0.0001.

Imiquimod (R837) and resiquimod (R848) are synthetic imidazoquinoline TLR7 site 1 ligands that bind independently of ssRNA (33). To determine if transcriptional alterations triggered by PNPi with guanosine nucleosides overlap or diverge from those elicited by synthetic TLR7 ligands, we performed RNA-Seq analysis of BMDMs, following acute stimulation with PNPi+dG/rG, polyU, PNPi+dG/rG+polyU, or R848. Principal component analysis of significantly altered transcripts (>10 fold change, <0.01% FDR) revealed a significant separation among these groups (Figure 7A and Supplemental Data File 1). K-means clustering and ontology analysis of induced transcripts reveled that both TLR7 activation modalities elicited NF-κB–associated responses, but a type I IFN response was induced exclusively by ssRNA and PNPi-stabilized guanosine ligands (Figure 7, B and C). A titration of R848 confirmed that, unlike endogenous coligands, this synthetic TLR7 agonist does not induce type I IFN expression in BMDMs (Figure 7D). These findings indicate that PNP tunes TLR7-driven transcriptional responses to ssRNA and reveal that natural or synthetic TLR7 ligands elicit distinct transcriptional profiles in BMDMs.

### PNP is a metabolic immune checkpoint that controls germinal center formation and autoimmunity.

**Figure 8:**
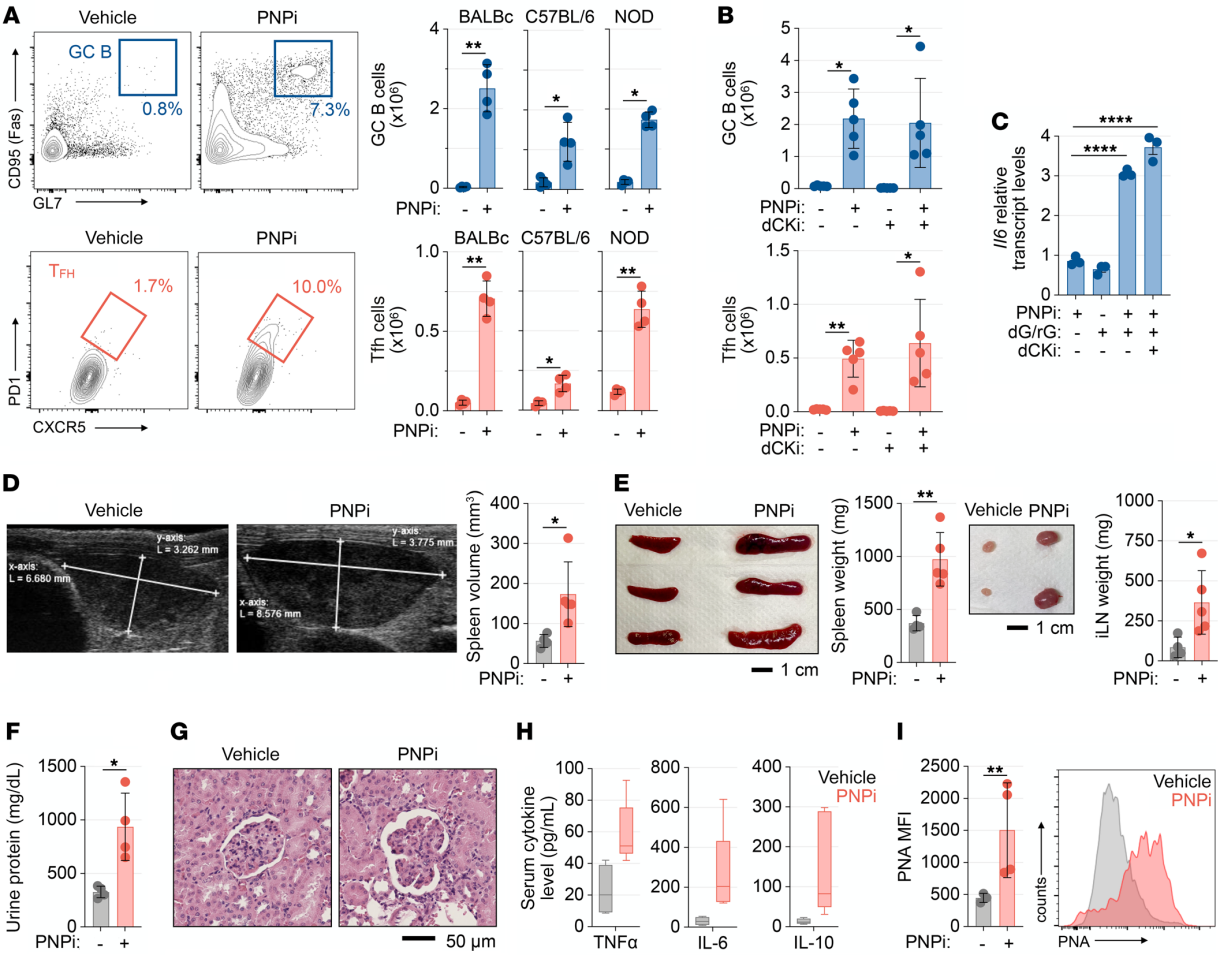
PNP inhibition promotes the germinal center reaction and accelerates autoimmunity. (**A**) Flow cytometry analysis of spleen germinal center (GC) B cell and T follicular helper (Tfh) cell abundance in female BALB/c, C57BL/6, and NOD mice treated with or without 100 mg/kg ulodesine (PNPi; p.o.; q.d.) for 14 days (*n =* 4/group; 2-tailed Mann-Whitney test). (**B**) Spleen GC B cell and Tfh cell abundance in BALB/c mice treated with or without 100 mg/kg PNPi (q.d.) and 100 mg/kg (*R*)-DI-87 (dCKi; q.d.) for 14 days (*n =* 5/group; Kruskal-Wallis test with Dunn’s multiple comparisons correction). (**C**) RT-PCR analysis of C57BL/6 CD43^–^ B cells stimulated with or without 1 μM PNPi, 5 μM dG/rG, and 1 μM dCKi in vitro for 4 hours (mean ± SD; *n =* 3; 1-way ANOVA corrected for multiple comparisons). (**D**) Analysis of female MRL-LPR mice treated with vehicle or PNPi ad libitum (20 mg/kg daily) for 35 days. Representative spleen ultrasound images and calculated volumes from day 35 analysis (mean ± SD; *n =* 4 vehicle; *n =* 5 PNPi; unpaired *t* test). (**E**) Analysis of spleens and inguinal lymph nodes (iLNs) from MRL-LPR mice treated with or without PNPi (mean ± SD; *n =* 4 vehicle; *n =* 5 PNPi; unpaired t test). (**F**) Total urine protein levels from MRL-LPR mice treated with or without PNPi (mean ± SD; unpaired *t* test). (**G**) Representative kidney H&E immunohistochemistry analysis of MRL-LPR mice treated with or without PNPi. Scale bar: 50 μm. (**H**) Luminex serum cytokine analysis of MRL-LPR mice treated with or without PNPi. (**I**) Flow cytometry analysis of splenic CD19^+^ B cells from MRL-LPR mice treated with or without PNPi (mean ± SD; *n =* 3–4; unpaired *t* test). *P < 0.05; **P < 0.01; ****P < 0.0001.

Our studies indicated that PNP inhibition triggers TLR7 activation in B cells both in vivo and in cell culture (Figure 5H**)** and Supplemental Figure 5E). B cell–intrinsic TLR7 signaling and IL-6 production promote the development of spontaneous germinal centers (GCs) in secondary lymphoid tissues, which are associated with autoinflammation and autoimmunity (31, 36). To investigate a role for PNP in regulating the GC reaction, we evaluated C57BL/6, BALB/c, and NOD mice treated with PNPi daily for 2 weeks. Splenomegaly and increased spleen cellularity were observed in all treated mice (Supplemental Figure 6A). PNPi stimulated the expansion of the CD19^+^GL7^+^CD95^+^PNA^+^ GC B cells in the spleen and inguinal lymph nodes (Figure 8A and Supplemental Figure 6, B and C). PNPi also significantly increased the abundance of GC-participating TCRβ^+^CD4^+^CD62L^–^CXCR5^+^PD1^+^ T follicular helper cells. Moreover, the expression of the GC-promoting cytokine IL-6 increased in splenic B cells from BALB/c mice following PNPi treatment (Supplemental Figure 6D).

Unlike the T cell developmental defects induced by PNP inhibition (Figure 1B), the effects of PNPi treatment on secondary lymphoid organs and GC formation were independent of dCK activity, as dCKi coadministration did not effect PNPi-induced alterations in spleen weight, GC B cell, or T follicular helper cell expansion (Figure 8B). Moreover, dCK inhibition did not affect *Il6* upregulation by PNPi+dG/rG in B cells in vitro (Figure 8C). Together, these results indicate that (a) PNP inhibition in mice triggers the formation of spontaneous GCs in the absence of foreign antigen and (b) dCK/SAMHD1- and TLR7-dependent effects of PNP inactivation can be functionally uncoupled by dCK inhibition, which restricts PNP inactivation-induced dGTP pool expansion while retaining TLR7-driven cytokine responses.

Constitutive TLR7 signaling is associated with the development of systemic lupus erythematosus–like autoimmune phenotypes in mice and humans (36). Additionally, various autoimmune pathologies are characteristic of PNP deficiency in humans (6). To determine if PNP inactivation in mice engenders similar autoimmunity-associated phenotypes we used MRL-LPR mice, which are prone to the development of systemic autoimmunity and B cell–dependent nephritis (37). We treated MRL-LPR mice with PNPi for 35 days and used ultrasound imaging to monitor increases in spleen size as an indicator of disease progression. PNPi-induced splenomegaly was apparent as early as day 22 and was sustained until the experimental endpoint (Figure 8D and Supplemental Figure 6E). This finding was corroborated by measurements of spleen mass (Figure 8E). PNPi treatment also increased serum cytokine levels and accelerated the onset of other manifestations of autoimmunity in the MRL-LPR model, including lymphadenopathy, proteinuria, and glomerulonephritis (Figure 8, E–H, and Supplemental Figure 6, F and G). Activation of splenic B cells by PNPi was evidenced by increased PNA binding in CD19^+^ cells (Figure 8I).

These findings indicate that PNP functions as a metabolic immune checkpoint that restricts inappropriate TLR7 activation, controls the formation of spontaneous GCs, and participates in the maintenance of B cell tolerance.

## Discussion

Here we provide mechanistic insight into the immunoregulatory functions of PNP and guanine nucleosides. We show that, in developing thymocytes, PNP functions to prevent severe dNTP pool imbalances caused by excessive production of dGTP via dCK-dependent dG salvage. Unlike other immune lineages, thymocytes cannot compensate for PNP inactivation through SAMHD1-mediated phosphohydrolysis of excess dGTP. The synthetic lethal interaction between PNP and SAMHD1 inactivation explains one of the enduring mysteries regarding the mechanisms responsible for T cell immunodeficiency in patients with PNP-inactivating mutations.

We also explain why genetic or pharmacologic PNP inactivation in mice does not recapitulate the severe T cell immunodeficiency observed in humans. dC, which is present at much higher levels in mice than in humans and nonhuman primates, competes with dG for dCK-mediated phosphorylation. This protective mechanism is attenuated in BLT-humanized mice which display an increased rate of dC catabolism and possess low serum dC levels approaching those measured in humans and nonhuman primates. Consequently, PNP inhibition in humanized mice significantly affects T cell development, indicating that these mice provide an appropriate model for alterations in thymocyte nucleotide metabolism caused by PNP deficiency. However, differences in the severity of the immunodeficiency between PNP-deficient patients and mice might also be due to exposure, as immune-deficient mice are typically housed in pathogen-free or controlled environments.

We show that PNP functions as a potentially novel metabolic immune checkpoint that regulates TLR7 signaling by restricting the availability its site 1 (deoxy)guanosine ligands. Overriding this checkpoint promotes TLR7 activation, triggers spontaneous GC responses in the absence of exogenous antigen, and accelerates the onset of autoimmunity-linked phenotypes in MRL-LPR mice. These findings explain another longstanding question regarding the mechanism responsible for the autoimmune and inflammatory manifestations frequently associated with PNP deficiency in patients. Furthermore, they provide a potential explanation for pancytopenia and massive splenomegaly that occur in PNP-KO mice that appears to be responsible for their reduced life span. Beyond altered B cell function, the increased levels of cytokines, such as IL-6 and TNF-α observed in PNPi-treated mice, may corrupt the functions of regulatory T cells and may also contribute to the observed autoimmune manifestations (38).

While our findings shed light on the complex metabolic and signaling alterations associated with PNP deficiency, they also raise new questions concerning the regulation of SAMHD1 expression and the mechanisms that modulate inappropriate TLR7 activation by PNP inhibition. SAMHD1 limits dNTP abundance, restricts endogenous retro-elements, and maintains replication fork integrity during S phase (39, 40). The mechanism of SAMHD1 downregulation in thymocytes and whether one or more of these functions negatively affects T cell development requires further investigation.

Regarding TLR7 activation, recent studies show that uridine-containing ssRNA and uridine/guanine nucleosides are produced from pathogen-derived RNA simultaneously within endolysosomes by RNASET2-mediated breakdown in macrophages (41). While PNP appears to be excluded from endolysosomes, it may function to control endolysosomal guanine nucleoside levels in trans via its cytosolic or extracellular activity. Extracellular guanine nucleosides could enter endolysosomes via vesicle-mediated endocytosis/macropinocytosis or via plasma membrane and endosomal equilibrative nucleoside transporters SLC29A1/2 (ENT1/2) and SLC29A3 (ENT3) (42), respectively. PNP likely functions to intercept guanine nucleosides before they reach the endolysosomal compartment. PNP cytosolic activity could also drive a concentration gradient that promotes the efflux of nucleosides from endolysosomes via ENT3. PNP thereby increases the threshold for TLR7 activation by pathogen or self RNA-derived guanine nucleosides. Functional evidence of a role for guanosine in regulating TLR7 signaling in humans was provided by the identification of a gain-of-function TLR7 mutation that enhances guanosine ligand aﬃnity and promotes autoimmunity (43). This report and our results together indicate that decreasing the threshold for guanosine nucleoside sensing by TLR7, either via sustained inhibition of PNP-mediated guanosine breakdown or a gain-of-function mutation, promotes GC formation and autoimmunity.

Our findings are also important from a translational perspective. While allogeneic hematopoietic stem cell transplantation is currently the only available treatment for PNP deficiency, our data suggest that combined pharmacological blockade of dCK and TLR7 signaling could represent an alternative therapeutic strategy. Our findings also suggest additional targeted clinical applications for current clinical-stage PNP inhibitors. Forodesine is approved in Japan for the treatment of PTCL and exhibits antitumor activity in a subset of patients, but it is unclear whether tumor cell lethality or TLR7 agonism underlies this effect (44). PNP inactivation is synthetically lethal with SAMHD1 deficiency; thus, PNPi may be particularly effective for the treatment of patients stratified by tumor cell SAMHD1 expression. PNP inhibitors additionally provide a novel approach for therapeutic TLR7 activation for use as a vaccine adjuvant (44) or for treatment of immunologically cold malignancies with upregulated PNP expression, such as pancreatic cancer (45). Compared with synthetic small molecule agonists, such as R837 or R848, which are not amenable to repetitive dosing secondary to toxicity, PNP inhibitors are well tolerated and trigger unique transcriptional responses.

Beyond the control of nucleoside levels, PNP enables a parallel salvage route to the de novo pathway for purine synthesis by catalyzing the release of hypoxanthine and guanine nucleobases, which can be recaptured into nucleotide pools by HPRT. Clinical evidence indicates that loss of purine salvage biosynthesis capability may be detrimental to particular cell types with a heightened energy state, including midbrain dopaminergic cells, as inactivation of HPRT causes Lesch-Nyhan syndrome, a severe neurological disorder (46). Whether impaired purine salvage or other effects, such as dysregulation of TLR7 activity in microglia, cause the described neurological manifestations of PNP inactivation remains to be determined (47).

### Limitations of the study.

Our findings underscore the importance of considering metabolic and immune differences between mice and humans in the design of translational research studies. Interspecies differences can be addressed through the development of mouse models that possess human nucleoside levels without the inherent limitations of the BLT mice, which are not readily amenable for genetic studies of T cell development. Our data offer guidance on future directions; one possibility is the development of mice that possess humanized systemic CDA activity. Regarding the role of PNP as a negative regulator of TLR7 signaling, the lack of functional TLR8 expression in mice (48) may confound the autoimmune and inflammatory phenotypes caused by PNP inhibition. Future studies in humanized TLR8-expressing mice (49) will be required to address this limitation.

## Methods

### Cell culture

Cell culture was performed as previously described (50). All cancer cell cultures were maintained between passages 3 and 20 and maintained in antibiotic-free media plus 10% FBS at 37°C in 5% CO_2_. All leukemia and lymphoma models were maintained in RPMI plus 10% FBS. Primary T cell cultures were maintained in RPMI with 10% FBS and 30 U/mL recombinant IL-2. Primary B cell cultures were maintained in RPMI with 10% FBS and 1× sodium pyruvate and 0.1% β-mercaptoethanol. Solid tumor cultures were maintained in DMEM with 10% FBS. All cell culture experiments were performed using media supplemented with 10% dialyzed FBS. Cell cultures were routinely monitored for mycoplasma contamination using the PCR-based Venor Mycoplasma kit. Cell lines were acquired either from a commercial vendor (ATCC and DSMZ) or from collaborators. Cell line identity was independently authenticated by PCR (Laragen). Methods for culture and analysis of primary cells are included in Supplemental Methods.

### Drugs

Drugs for in vitro studies were prepared as previously described (50). Drug stocks were prepared in neat DMSO or H_2_O, stored at –80°C and diluted fresh in cell culture media for experiments. 21-mer polyU ssRNA was complexed with DOTAP before treatment.

### Animal studies

For treatment studies, ulodesine was formulated in 100% sterile distilled water and administered as indicated in a volume of 100 μL/20 g mouse; (*R*)-DI-87 was formulated in 40% captisol and administered at 75 mg/kg p.o. b.i.d. or 100 mg/kg p.o. q.d. in a volume of 100 μL/20 g mouse. For ad libitum administration of PNPi, ulodesine was formulated in drinking water at 0.13 mg/mL, which was intended to deliver a daily dose of 20 mg/kg. Urine protein was measured using the BCA assay (Supplemental Table 1).

For subcutaneous tumor models, 6- to 8-week-old male NOD-Prkdc^em26Cd52^;Il2rg^em26Cd22^;NjuCrl coisogenic immunodeficient (NCG) mice were injected subcutaneously on the flanks with 1 × 10^6^ CEM cells resuspended 1:1 in PBS/Matrigel (50 μL/50 μL). Subcutaneous tumor volumes were calculated by micro-computed tomography (μCT) analysis using a G8 PET/CT scanner. All tumor volume measurements were performed by trained technicians blinded to experimental conditions.

NSG and TKO BLT-humanized mice were derived by the UCLA CFAR Humanized Mouse Core Facility. The generation of these mice has been previously described (51). For these mice, human fetal liver and autologous thymus fragments were coimplanted under the renal capsule of NSG or TKO mice and human HSCs were engrafted to the bone marrow by intravenous injection. Human lymphocyte reconstitution was evaluated by flow cytometry analysis of percentage of human CD45-reactive peripheral blood cells and confirmed to be more than 30% for all mice before enrollment for treatment studies. Humanized mouse treatment studies were initiated 4 months after tissue engraftment.

Ultrasound imaging was performed using the Vevo 2100 Ultrasound from Visual Sonics equipped with an ultrahigh frequency linear probe. Prior to imaging, animals underwent hair removal with clippers and depilatory cream. 3D scans were obtained and exported for analysis using the companion software, Vevo Lab version 5.5.1. Spleen volume measurements were performed using the multislice setting and a step size factor of 1 and were reported in mm^3^.

### ATO model

#### Isolation of human cord blood HSPCs for ATO culture.

Neonatal umbilical cord blood was obtained from discarded cord and placental units from deliveries at UCLA and was enriched for mononuclear cells by Ficoll-Paque gradient centrifugation, followed by positive selection of CD34^+^ cells by magnetic cell sorting using the CD34 MicroBead UltraPure Kit (Supplemental Table 1). CD34^+^ enriched fractions were cryopreserved after MACS. Prior to use, cells were thawed, and residual T cells were depleted by FACS by sorting for CD34^+^CD3^–^ cells. Sorted cells were immediately seeded into MS5-hDLL4 ATOs, as previously described (14).

#### Cord blood HSPC ATO cultures.

Cord blood ATOs were generated as previously described (14). MS5-hDLL4 cells were harvested by trypsinization and resuspended in serum-free ATO culture medium (RB27) composed of RPMI with 4% B27 supplement and 30 mM L-ascorbic acid 2-phosphate sesquimagnesium salt hydrate reconstituted in PBS with 1% penicillin/streptomycin and 1% Glutamax, 5 ng/ml rhFLT3L, and 2.5 ng/ml rhIL-7. 1.5 × 10^5^ MS5-hDLL4 cells were combined with 5 × 10^3^ purified CD34^+^CD3^–^ cells per ATO in 1.5 mL Eppendorf tubes and centrifuged at 300*g* for 5 minutes at 4°C in a swinging bucket centrifuge. Supernatants were carefully removed, and the cell pellet was resuspended in 6 μL RB27 per ATO and mixed by brief vortexing. ATOs were plated on a 0.4 mm Millicell Transwell insert placed in a 6-well plate containing 1 mL RB27 per well with the small molecules as indicated. Medium was changed completely every 3–4 days by aspiration from around the cell insert, followed by replacement with 1 mL with fresh RB27/cytokines/small molecules.

### RNA-Seq

#### Sample preparation.

BMDM cells were seeded overnight and treated as indicated. Following treatment, mRNA was extracted as described for RT-PCR analysis and processed for next-generation sequencing.

#### Library construction and next-generation sequencing.

Libraries for RNA-Seq were prepared with the KAPA Stranded mRNA-Seq Kit (Supplemental Table 1). The workflow consisted of mRNA enrichment and fragmentation, first-strand cDNA synthesis using random priming, followed by second-strand synthesis converting cDNA/RNA hybrid to double-stranded cDNA, and incorporating dUTP into the second cDNA strand. cDNA generation was followed by end repair to generate blunt ends, A-tailing, adaptor ligation, and PCR amplification. Different adaptors were used for multiplexing samples in 1 lane. Sequencing was performed on an Illumina HiSeq 3000 (1 × 50 bp read length). Data quality checking was performed on an Illumina SAV. Data demultiplexing was performed with Illumina Bcl2fastq v2.19.1.403 software.

#### RNA-Seq bioinformatics analysis.

Reads were mapped by STAR 2.27a, and read counts per gene were quantified using the mouse Ensembl Mus_musculus.GRCm38.97 GTF file. In Partek Flow, read counts were normalized by CPM + 1.0 × 10^–4^, and differential expression analysis was performed via ANOVA. For differentially expressed gene lists, *P* value, FDR, and fold change filters were applied. A *P* value cutoff of *P* < 1 × 10^–5^ was applied to identify which genes showed evidence of differential expression in at least 1 comparison group. The FDR and fold change cutoffs were then set at FDR < 1 × 10^–3^ and fold change less than 10 to determine the topmost differentially expressed genes. Principal component analysis and K-means clustering were performed using Phantasus. For pathway analysis, enrichment of cluster-defining genes across MSigDB (https://www.gsea-msigdb.org/gsea/msigdb/) 2020 hallmark ontologies was performed. Raw and analyzed data were deposited in NCBI GEO (GSE203003).

### scRNA-Seq

Methods for scRNA-Seq data acquisition and analysis are included in Supplemental Methods.

### Immunoblot analysis

PBS-washed cell pellets obtained by trypsinization of cells and centrifugation at 450*g* for 5 minutes were resuspended in cold RIPA buffer supplemented with protease and phosphatase inhibitor cocktails. Sample protein content was determined using the BCA assay. Samples were normalized by RIPA and 4× Laemmli loading dye dilution, resolved on 4%–12% Bis-Tris gels, and electrotransferred to nitrocellulose membranes. After blocking with 5% nonfat milk in TBS 0.1% Tween-20 (TBS-T) for 30 minutes at room temperature, membranes were incubated overnight in primary antibodies diluted 1:1000 in 5% BSA in TBS-T at 4°C. Membranes were washed with TBS-T and incubated with HRP-linked secondary antibodies prepared at a 1:2500 dilution in 5% nonfat dry milk/TBS-T for 1 hour at room temperature. HRP was activated by incubating membranes with a mixture of SuperSignal Pico and SuperSignal Femto ECL reagents (33:1 ratio). Exposure of autoradiography film was used for detection. Antibodies are reported in Supplemental Table 1. Catalog information for all reagents is included as Supplemental Table 1. See complete unedited blots in the supplemental material.

### RT-PCR

RT-PCR was performed as previously described (50). Total RNA was isolated from cell cultures using the NucleoSpin RNA kit (Supplemental Table 1) and quantified by Nanodrop. Total RNA was isolated from 30 to 80 mg of snap frozen tissues by homogenization in 500 μL extraction buffer in Omni Hard Tissue homogenization vials using an Omni Bead Ruptor Elite (8 cycles of 15 seconds on, 30 seconds off, speed 8) chilled to 4°C, and isolated using the NucleoSpin RNA kit. Reverse transcription was performed using the High Capacity cDNA Reverse Transcription kit (Supplemental Table 1). Quantitative PCR was performed using EvaGreen qPCR Master Mix on the QuantStudio3 system (Supplemental Table 1). RNA expression values were normalized to housekeeping gene (ACTIN, Actb) expression, calculated using the ^ΔΔ^Ct method, and reported as relative expression to vehicle control-treated samples. Primer sequences are indicated in Supplemental Table 1.

### Cell proliferation analysis

For Cell Titer Glo (Supplemental Table 1) analysis, cells were seeded at 1 × 10^3^ cells per well in 50 μL media with 10% dialyzed FBS in white 384-well plates and treated as described. After 72 hours, 50 μL Cell Titer Glo reagent (diluted 1:5 in PBS) was added to each well and luminescence was measured using a Synergy H1 reader (Supplemental Table 1).

### Generation of isogenic cell line models

#### Stable expression models.

Derivation of stable-expressing isogenic cells was performed as previously described (50). A yellow fluorescent protein (YFP) control or human SAMHD1-encoding gene fragment was ligated into the pENTR-D/TOPO entry vector. The resulting constructs were recombined into pLenti-CMV/TO-GFP/PURO using Gateway LR Clonase II. For virus production, lentiviral vectors and packaging plasmids (psPAX2, pMD2G) at a 2:1:1 ratio were transfected into FT293 cells using polyethylenimine. Transduced cells were selected in puromycin for 1 week. The generation of a CEM model stably expressing CDA was previously described (30).

#### CRISPR/Cas9 gene KO.

All gRNA-encoding oligonucleotides were cloned into the LentiCrisprV2 vector. Lipofectamine 3000 was used to transfect PDAC cells with gRNA-specific LentiCrisprV2 vectors. Following puromycin selection, cells were singly cloned and gene KO was confirmed by genomic DNA PCR/TIDE in/del analysis of Sanger sequencing results. Gene KO was additionally validated using immunoblot analysis.

### Luminex analysis

Blood was collected in 1.5 mL Eppendorf tubes by the retro-orbital technique using heparin-coated capillary tubes and incubated at room temperature for 30 minutes. Samples were centrifuged at 2000*g* for 15 minutes at 4°C, and serum was transferred to a new tube for 32-plex Luminex analysis performed by the UCLA Immune Assessment Core.

### Flow cytometry

Methods for flow cytometry data acquisition and analysis are included in Supplemental Methods.

### Mass spectrometry

Methods for mass spectrometry data acquisition and analysis are included in Supplemental Methods.

### Statistics

Statistical analysis was performed as previously described (50). Data are presented as mean ± SD, with the number of biological replicates indicated. Comparisons of 2 groups were evaluated using either unpaired 2-tailed Student’s *t* test or 2-tailed Mann-Whitney test, and *P* values of less than 0.05 were considered significant. Comparisons of more than 2 groups were evaluated using either 1-way ANOVA, followed by Bonferroni’s multiple comparisons test or Kruskal-Wallis test with Dunn’s multiple comparisons correction, and *P* values less than 0.05/*m*, where *m* is the total number of possible comparisons, were considered significant.

### Study approval

All animal studies were approved by the UCLA Animal Research Committee.

Neonatal umbilical cord blood was obtained under UCLA IRB-approved protocols or exemptions.

## Author contributions

ERA, KR, TML, and CGR designed the study. ERA, KR, VL, and ALC performed research. ERA, KR, AC, ANL, HKM, VR, and NW performed animal studies. KR derived primary cell models. SL performed ATO studies. TML performed mass spectrometry analysis. HRL and WH performed scRNA-Seq analysis. LL generated cell line models. AKL, GAR, SDM, TM, SB, AR, TRD, GMC, and TTW contributed critical reagents and provided experimental support. EWR revised the manuscript. ERA, KR, TML and CGR analyzed data. ERA prepared figures. ERA and CGR wrote the manuscript.

## Supplementary Material

Supplemental data

Supplemental data set 1

## Version 1 06/02/2022

In-Press Preview

## Version 2 08/15/2022

Electronic publication
