# Supplementary material for: Purine nucleoside phosphorylase enables dual metabolic checkpoints that prevent T cell immunodeficiency and TLR7-associated autoimmunity
Source: J Clin Invest. 2022 Aug 15;132(16):e160852. doi: 10.1172/JCI160852 (PMC9374381; doi:10.1172/JCI160852)
Supplement: Supplemental data [file jci-132-160852-s117.pdf]

Supplemental Figure 1
Supporting Data for Figure 1

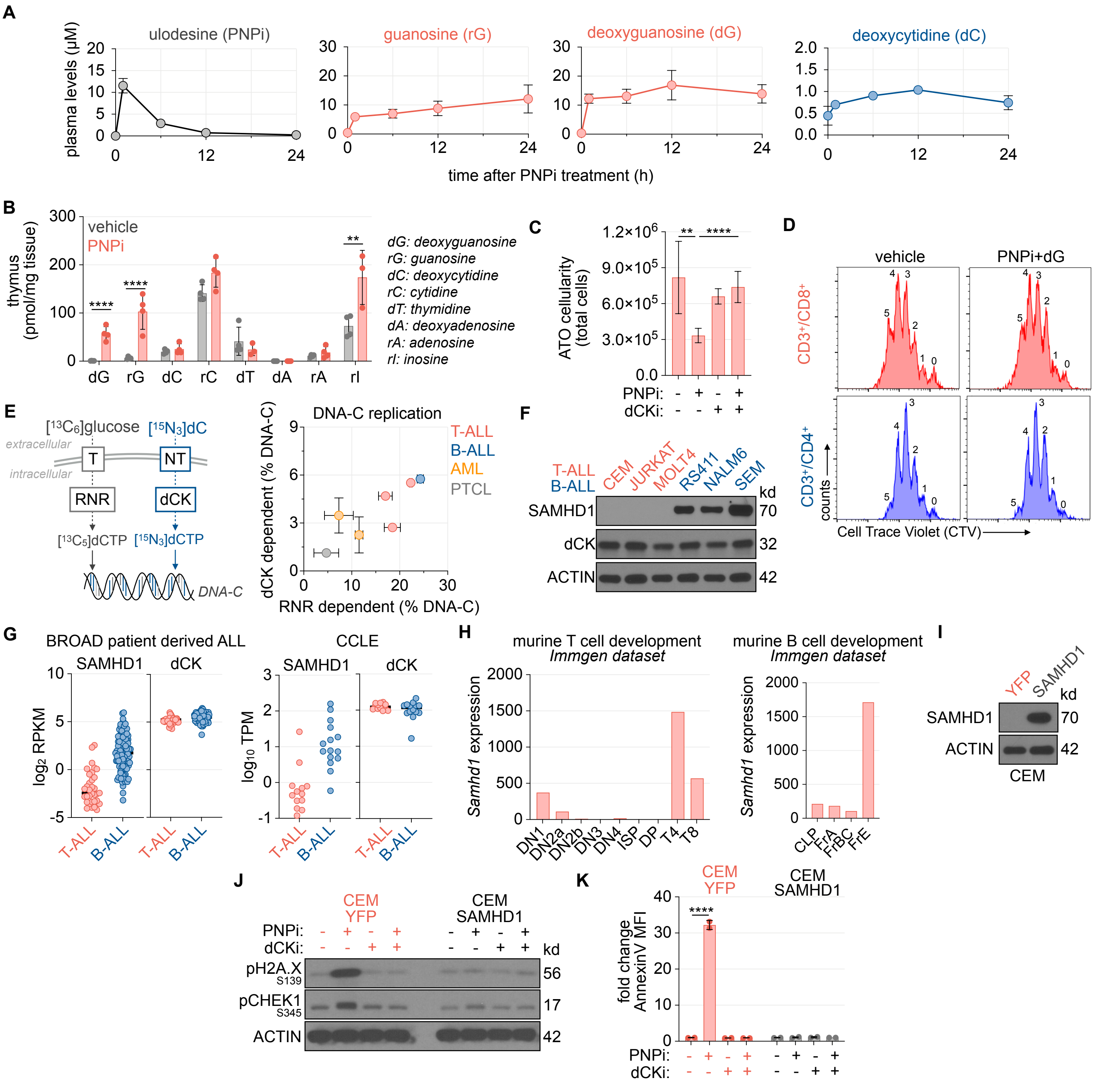

Figure S1 | Cell-autonomous dCK and SAMHD1 activity determines PNPi lethality. Related to Figure 1. (**A**) LC-MS/MS-MRM analysis of NCG mouse serum obtained at indicated timepoints following treatment + 100 mg/kg ulodesine (PNPi; p.o.; n=3). (**B**) LC-MS/MS-MRM analysis of thymus metabolite composition in C57BL/6 mice 24 h after treatment + 100 mg/kg PNPi (p.o.) or vehicle (mean±SD; n=4; unpaired t-test). (**C**) Quantification of artificial thymic organoid (ATO) cellularity at week 5 following continuous treatment + 5 µM deoxyguanosine (dG) ± 1 µM PNPi ± 1 µM (*R*)-DI-87 (dCKi; mean±SD; n=12; one-way ANOVA corrected for multiple comparisons). (**D**) Flow cytometry analysis of $CD3^+/CD8^+$ or $CD3^+/CD4^+$-gated C57BL/6 mouse splenocytes pulsed with Cell Trace Violet (CTV) and stimulated *in vitro* + 5 µM dG ± 1 µM PNPi for 72 h. Division numbers are indicated. (**E**) LC-MS/MS-MRM analysis of RNR dependent and dC salvage pathway contribution to DNA deoxycytidine (DNA-C) in leukemia cell lines. Cells were cultured with 1 g/L [$^{13}C_6$]glucose (to track RNR dependent *de novo* nucleotide synthesis) and 5 µM [$^{15}N_3$]dC (to track dCK dependent salvage nucleotide synthesis) for 18 h before DNA extraction (mean ±SD; n=3; NT: nucleoside transporter). (**F**) Immunoblot analysis of human acute lymphoblastic leukemia (ALL) cell lines. (**G**) SAMHD1 and dCK expression in human cancer cell line encyclopedia (CCLE) T- and B-ALL models (Ghandi et al. Nature. 2019) and in human patient-derived T and B ALL cell lines (Broad). (**H**) SAMHD1 and dCK expression across murine T and B cell development from the Immgen dataset (Heng et al. Nat Immunol. 2008). (**I**) Immunoblot validation of CEM-YFP control and CEM-SAMHD1 cells. (**J**) CEM-YFP and CEM-SAMHD1 cells treated + 5 µM dG ± 1 µM PNPi ± 1 µM dCKi for 24 h. (**K**) Flow cytometry analysis of CEM-YFP and CEM-SAMHD1 cells + 5 µM dG ± 1 µM PNPi ± 1 µM dCKi for 24 h (mean±SD; n=3; one-way ANOVA corrected for multiple comparisons).

Supplemental Figure 2
Supporting Data for Figure 1

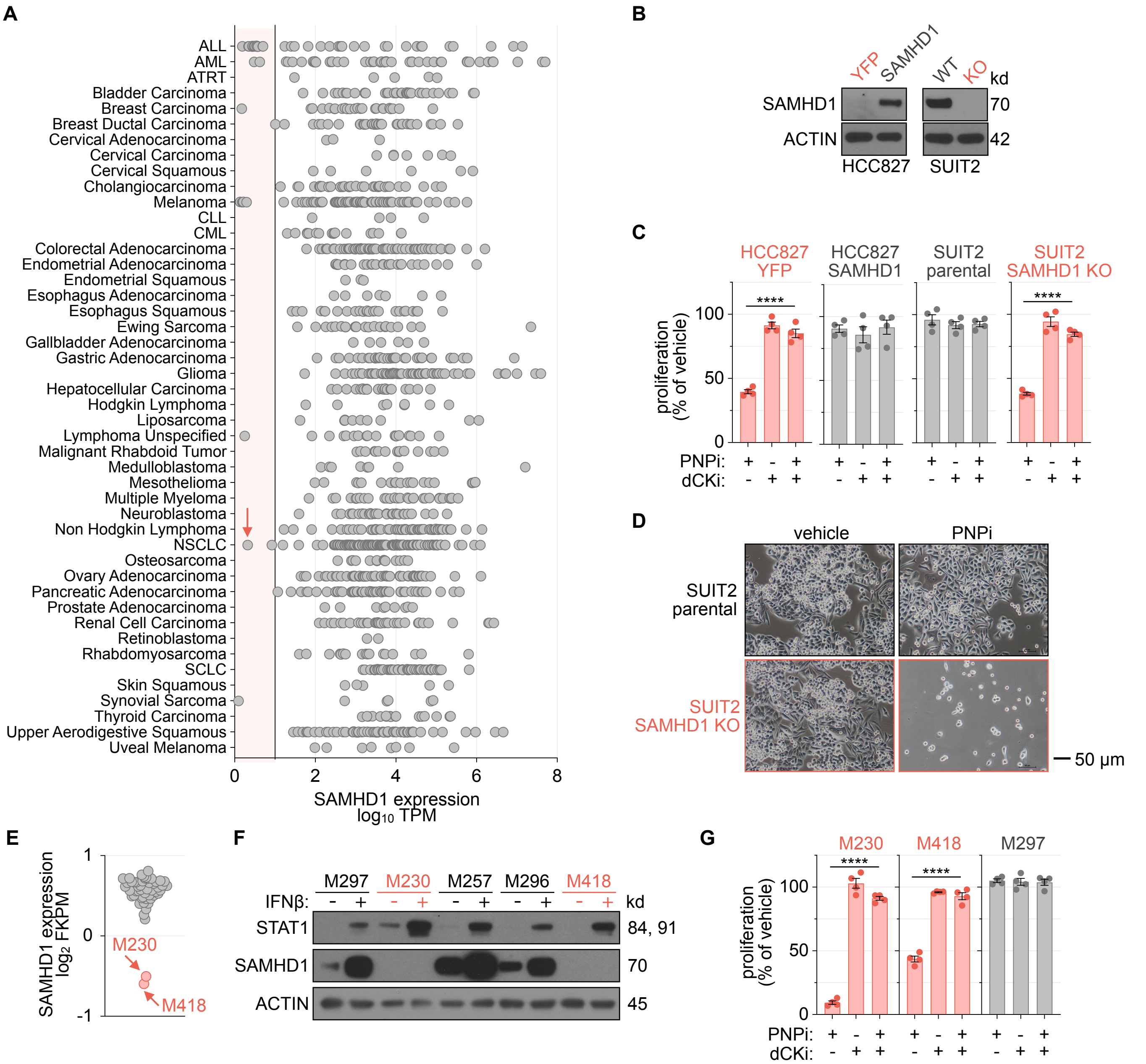

Figure S2 | SAMHD1 mediates PNPi lethality in solid tumor models. Related to Figure 1. (**A**) SAMHD1 expression across cancer cell line encyclopedia (CCLE) models (RNASeq; Ghandi et al. Nature. 2019). HCC827 is indicated. (**B**) Immunoblot validation of SUIT2 SAMHD1 CRISPR/Cas9 knockout (KO) and HCC827 SAMHD1-expressing isogenic cells. (**C**) Cell Titer Glo analysis of solid tumor isogenic cells treated + 5 µM dG ± 1 µM PNPi ± 1 µM dCKi for for 72 h (mean±SD; n=4; one-way ANOVA corrected for multiple comparisons). (**D**) Representative images of SUIT2 parental and SAMHD1 KO cells at 10x magnification following treatment + 5 µM dG ± 1 µM PNPi for 72 h. (**E**) RNAseq analysis of SAMHD1 expression in melanoma patient-derived xenograft (PDX) models. (**F**) Immunoblot analysis of melanoma PDX models treated ± 100 U/mL human IFNβ for 24 h. (**G**) Cell Titer Glo analysis of melanoma PDX models treated ± 1 µM PNPi / 5 µM dG ± 1 µM dCKi (mean±SD; n=4; one-way ANOVA corrected for multiple comparisons). | **** P<0.0001.

Supplemental Figure 3
Supporting Data for Figures 3 and 4

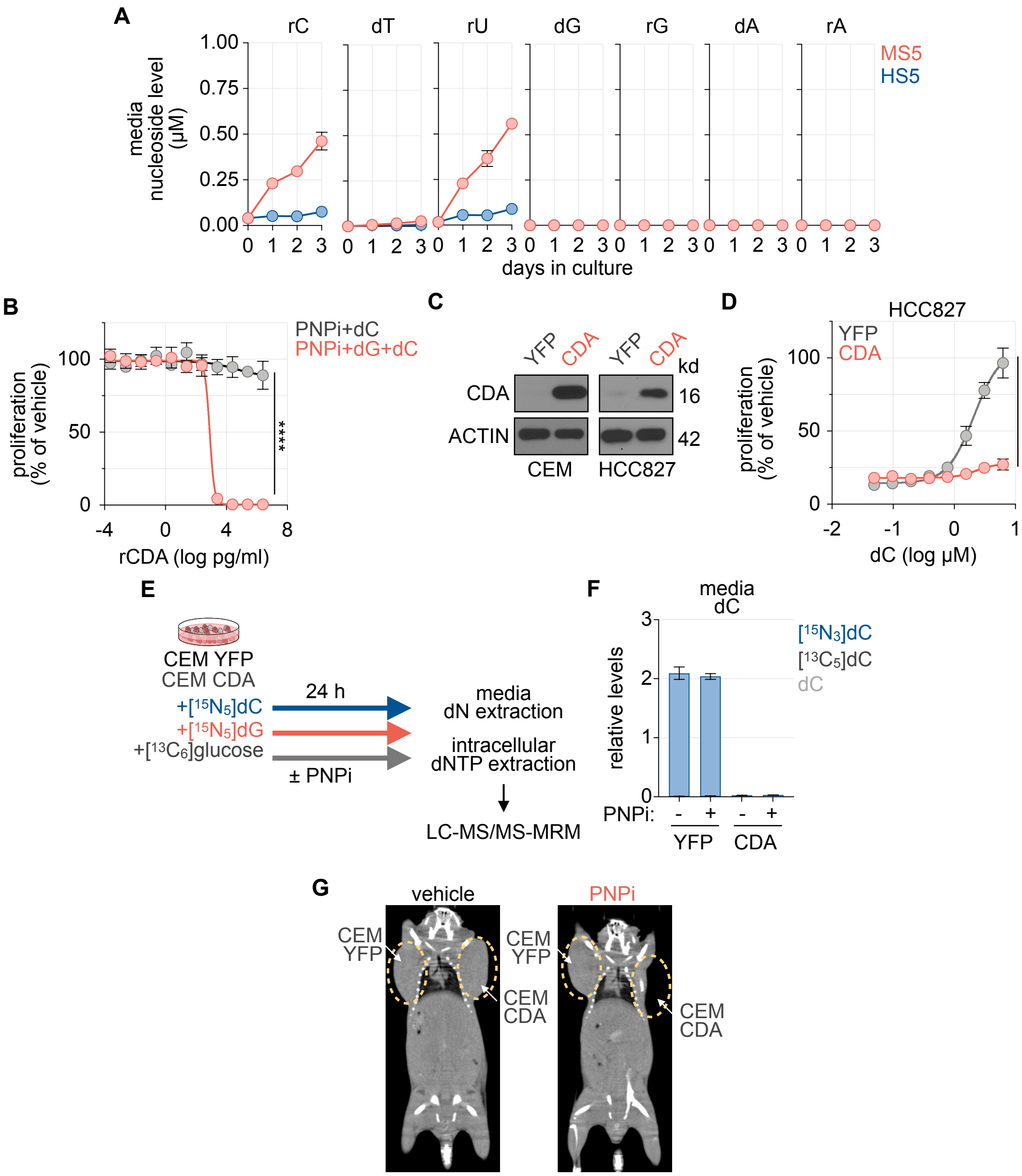

Figure S3 | dC mitigates PNPi lethality *in vitro* and *in vivo*. Related to Figures 3 and 4. (**A**) LC-MS/MS analysis of conditioned media (CM) derived from HS5 or MS5 cells from experiment in **Figure 3C** (mean ± SD; n=3). (**B**) Cell Titer Glo analysis of CEM-cells treated + 1 µM PNPi + 5 µM dG + 5 µM dC ± indicated recombinant cytidine deaminase (rCDA) for 72 h (n=4; mean±SD; unpaired t-test). (**C**) Immunoblot validation of CDA-expressing isogenic CEM and HCC827 cells. (**D**) Cell Titer Glo analysis of HCC827-YFP and -CDA cells treated + 1 µM PNPi + 5 µM dG ± a titration of dC for 72 h (n=4; mean±SD; unpaired t-test).(**E**) Experimental design for experiment in **Figure 4C**. (**F**) Media deoxycytidine measurements from endpoint of experiment in **Figure 4C**. (**G**) Representative µCT analysis from endpoint of experiment in **Figure 4D**. | *** P<0.001; **** P<0.0001.

Supplemental Figure 4
Supporting Data for Figure 5

A

cluster	vehicle	PNPi
**TOTAL CELLS**	**6701**	**6412**
B 1	1756	1146
B 2	235	636
B 3	370	457
B 4	320	265
B 5	85	131
B 6	80	68
B 7	61	39
B pre-mem	362	233
plasma cells	11	22
T/B doublet	79	50
CD8 T 1	691	671
CD8 T 2	224	237
CD4 T 1	1091	890
CD4 T 2	43	53
T_{reg}	180	207
Th1	78	73
NK	170	182
DC 1	156	172
DC 2	92	93
pDC	86	95
macrophage (Mk)/DC	316	297
macrophage (Mk)	69	188
monocyte/neutrophil	146	207

B

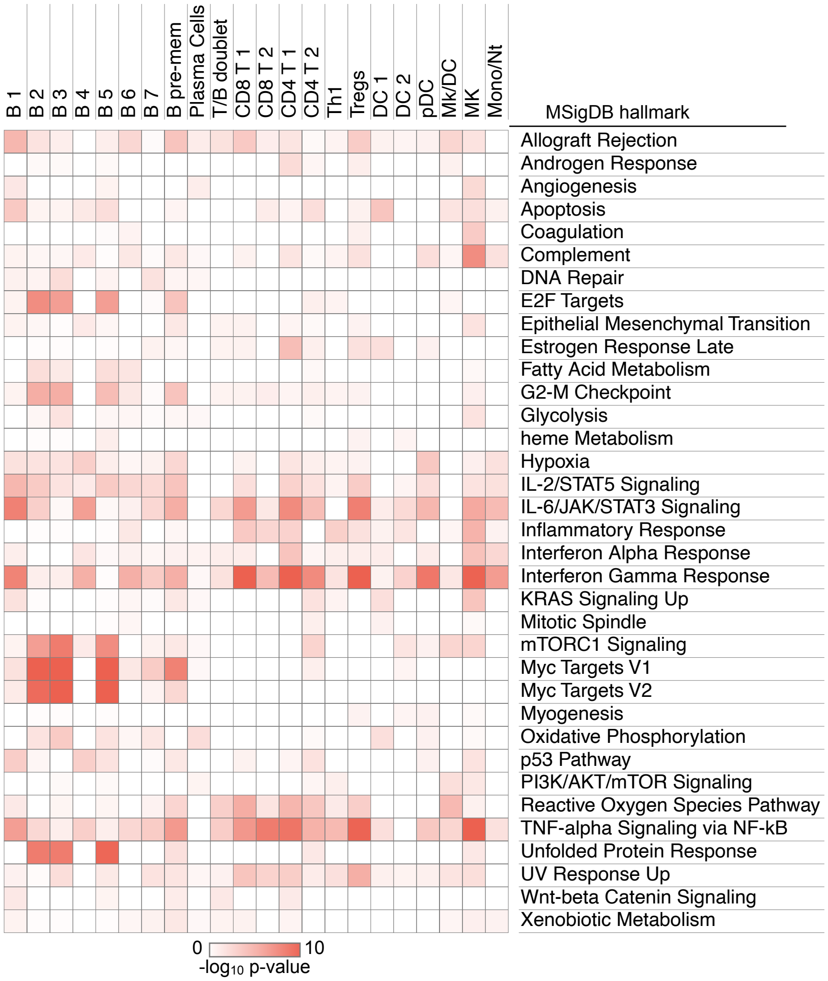

Figure S4 | scRNAseq analysis of spleen PNPi response. Related to Figure 5. (**A**) Summary of spleen scRNAseq cluster composition from experiment in **Figure 5E**. (**B**) Ontology analysis of significantly altered genes in clusters from experiment in **Figure 5E**.

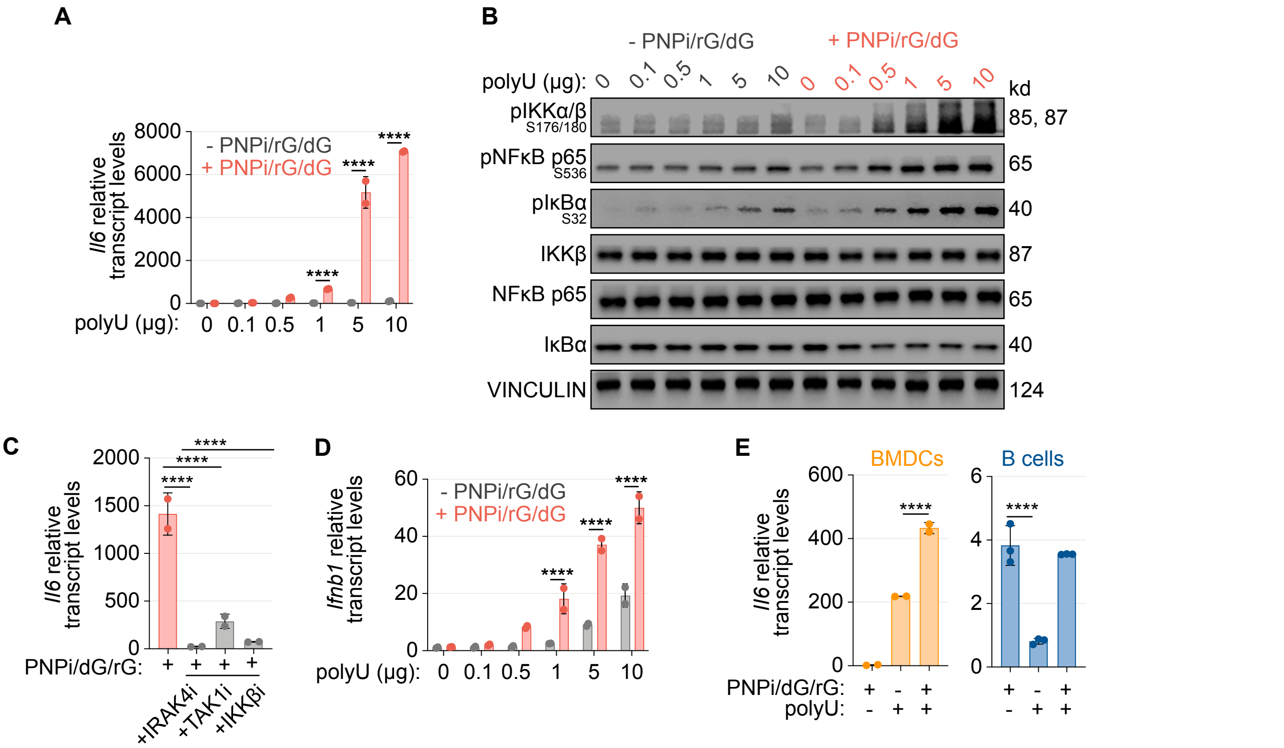

Figure S5 | PNPi activates TLR7 in BMDMs. Related to Figure 7. (**A**) RT-PCR analysis of BMDM treated ± 1 µM ulodesine (PNPi) / 5 µM guanosine (rG) and 5 µM deoxyguanosine (dG) ± polyU ssRNA (complexed with DOTAP) for 4 h (mean±SD; n=4; one-way ANOVA corrected for multiple comparisons). (**B**) Immunoblot analysis of BMDM treated ± 1 µM PNPi / 5 µM rG / 5 µM dG ± polyU (complexed with DOTAP) for 4 h. (**C**) RT-PCR analysis of BMDM treated ± 1 µM PNPi + 5 µM dG + 5 µM rG) / 5 µg polyU ± 1 µM PF-06650833 (IRAK4i) ± 10 µM takinib (TAK1i) ± 5 µM TPCA-1 (IKKβi) for 4 h (mean±SD; n=4; one-way ANOVA corrected for multiple comparisons). (**D**) RT-PCR analysis of BMDM treated ± 1 µM PNPi / 5 µM guanosine (rG) and deoxyguanosine (dG) ± polyU (complexed with DOTAP) for 4 h (mean±SD; n=4; one-way ANOVA corrected for multiple comparisons). (**E**) RT-PCR analysis of bone marrow-derived / FLT3 ligand-differentiated murine dendritic cell (BMDC) cultures and CD43^{-} murine splenocyte (B cell) cultures treated *ex vivo* ± 1 µM PNPi 5 µM 5 µM dG 5 µM 5 µM rG) ± 5 µg polyU ssRNA (complexed with DOTAP) for 4 h (mean±SD; n=4; one-way ANOVA corrected for multiple comparisons). | **** P<0.0001.

Supplemental Figure 6
Supporting Data for Figure 8

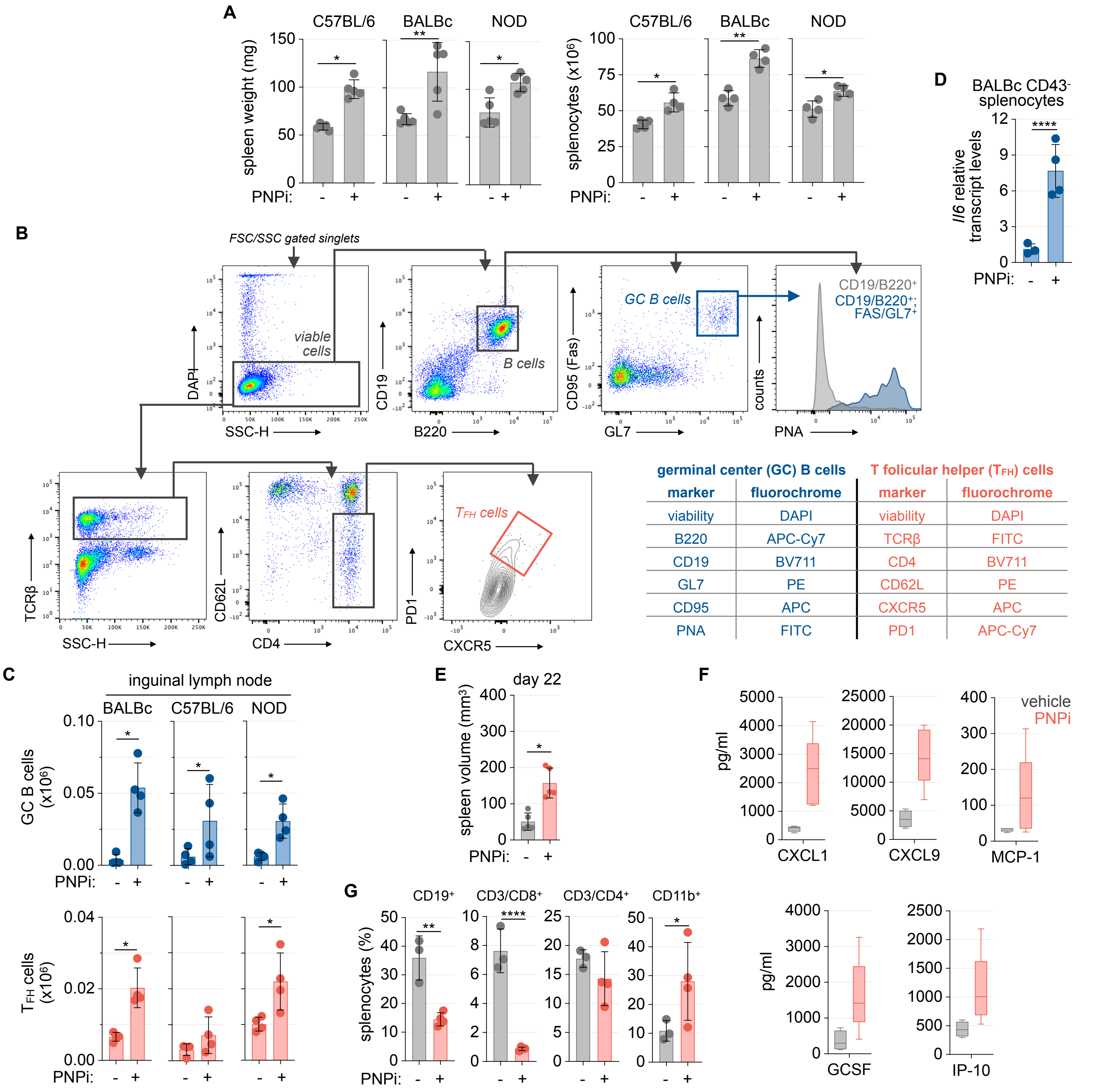

germinal center (GC) B cells		T follicular helper (TFH) cells	
marker	fluorochrome	marker	fluorochrome
viability	DAPI	viability	DAPI
B220	APC-Cy7	TCRβ	FITC
CD19	BV711	CD4	BV711
GL7	PE	CD62L	PE
CD95	APC	CXCR5	APC
PNA	FITC	PD1	APC-Cy7

Figure S6 | PNPi promotes germinal center formation and accelerates autoimmunity. Related to Figure 8. (**A**) Analysis of C57BL/6, BALBc and NOD mice treated ± 100 mg/kg ulodesine (PNPi; q.d.) for 14 d. Endpoint spleen weight and cellularity is indicated (n=4/group; two-tailed Mann-Whitney test). (**B**) Gating strategy and staining panel composition for the identification of germinal center (GC) B cells and T follicular helper (T_{FH}) cells by flow cytometry. (**C**) Quantification of PNPi induced alterations in total inguinal lymph node (iLN) GC B cell and T_{FH} abundance from BALBc, C57BL/6 mice and NOD mice treated ± 100 mg/kg PNPi (q.d.) from experiment in **Figure 8A** (n=4/group; two-tailed Mann-Whitney test). (**D**) RT-PCR analysis spleen-purified CD43- B cells from BALBc mice treated ± 100 mg/kg PNPi (p.o., q.d.) for 14 d from endpoint of experiment in **Figure 8A** (mean±SD; n=4/group; two-tailed Mann-Whitney test).(**E**) Spleen volume obtained by ultrasound evaluation at day 22 from experiment in **Figure 8E** (mean±SD; n=4 vehicle; n=5 PNPi; unpaired t-test) (**F**) Extended luminex cytokine analysis from endpoint of experiment in **Figure 8E**. (**G**) Flow cytometry analysis of spleen cellular composition in female MRL-LPR mice treated ± PNPi *ad. lib.* for 35 d (mean±SD; n=3 vehicle; n=4 PNPi). | * P<0.05; ** P<0.01; **** P<0.0001.

Immunoblot Source Data

Figure S1F

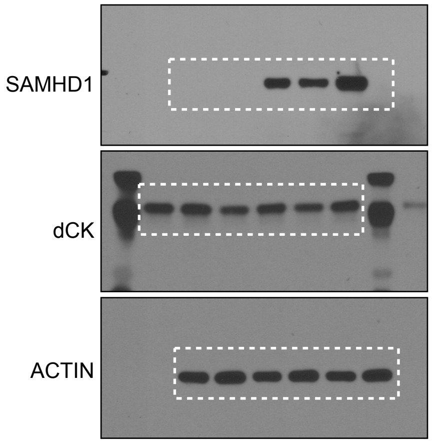

Figure S1I

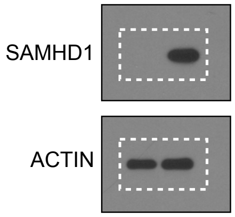

Figure S2B

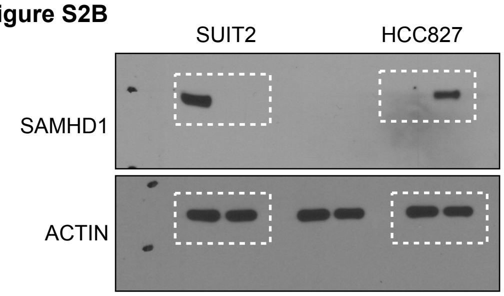

Figure S5B

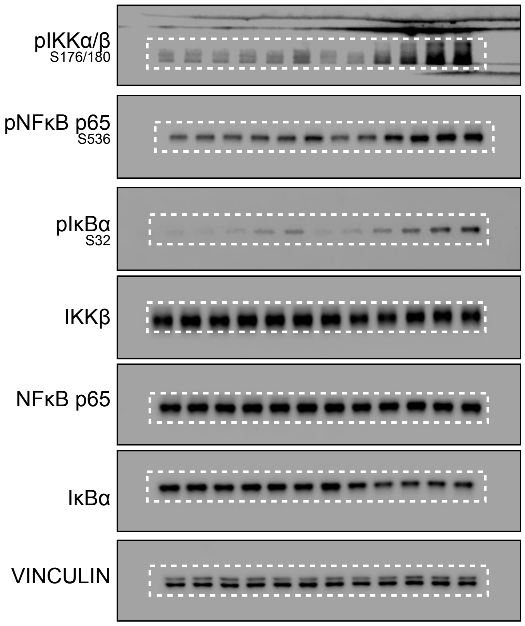

Figure S1J

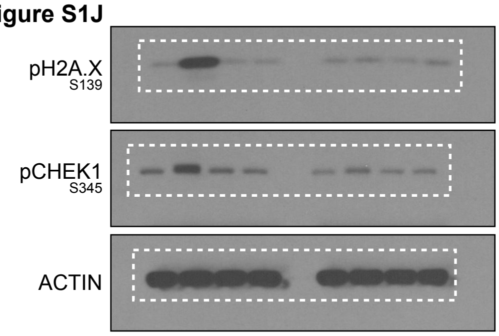

Figure S2F

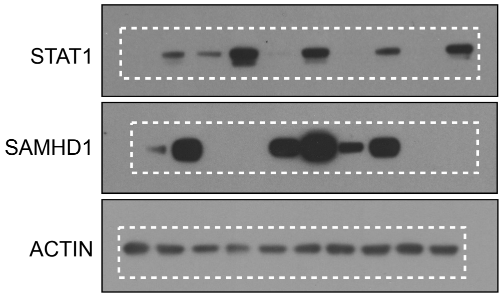

Figure 6I

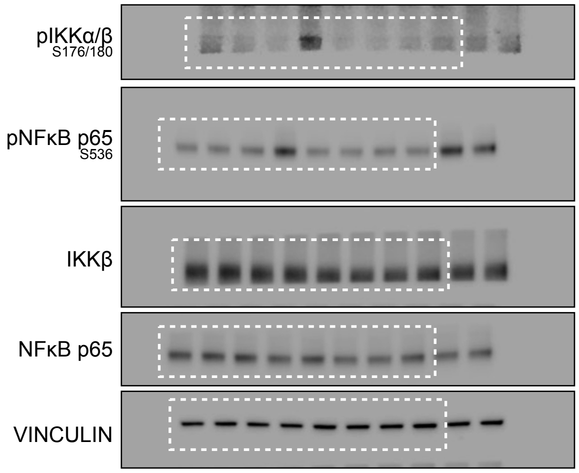

SUPPLEMENTAL METHODS

Primary Cell Culture

For the differentiation of murine bone marrow-derived macrophages (BMDM) or bone-marrow derived dendritic cells (BMDC), femur and tibiae were isolated from mice and stored on ice in PBS +5% FBS until processing. Bone marrow was flushed using a 25 gauge needle in 10 mL of PBS +5%FBS, centrifuged at 450xg for 4 minutes at 4°C, and resuspended in 2 mL of ACK lysis buffer. After 10 minutes of incubation, 2 mL of PBS +5%FBS was added to quench the reaction, suspensions were passed through a 70 µm nylon mesh filter and centrifuged at 450xg for 4 minutes at 4°C. For BMDM differentiation, bone marrow-derived cells were resuspended in 10 mL of DMEM +10%FBS +1x sodium pyruvate + 50 µM BME supplemented with 50 ng/mL M-CSF. For BMDC differentiation cells were resuspended in 10 mL of RPMI +10% FBS +1x sodium pyruvate +50 µM BME supplemented with 200 ng/mL FLT3L. 1x107 cells were plated in 10 cm plates and media was refreshed every 3 days. After 7 days of differentiation, cells were washed with PBS and collected by scraping with a rubber policeman in 5 mL of 10 mM EDTA in PBS. Cell lineage was confirmed by flow cytometry.

For the isolation of murine B lymphocytes, spleens from indicated mice were diced and mashed through a 70 µm nylon mesh strainer using a 1 mL syringe plunger in 10 mL of PBS +5% FBS buffer to obtain a single cell suspension. For red blood cell lysis, suspensions were centrifuged at 450xg for 4 minutes at 4°C, decanted, and cell pellets were resuspended in 3 mL of ACK lysis buffer and incubated for 3 minutes at room temperature. ACK was quenched by adding 10 mL of PBS +5% FBS buffer and passed through a 40 µm nylon mesh filter. $1x10^7$splenocytes were incubated with 10 µL of anti-CD43 MicroBeads in 100 µL of PBS +0.5% BSA +2mM EDTA (MACS buffer) for 15 minutes at 4°C. The cell suspension was passed through an MS column on an OctoMACS Separator and the flow-through (B cell fraction) was collected. The purity of the enriched fraction was evaluated by flow cytometry.

scRNA-seq

Sample preparation: Freshly isolated spleens from C57BL/6 mice 6 hours after treatment with 100 mg/kg ulodesine (PNPi; 100 µL p.o.) or vehicle (n=3 / group) were mashed through a 70 µm nylon mesh strainer using a 1 mL syringe plunger to obtain a single cell suspension. For red blood cell lysis, suspensions were centrifuged at 300xg for 2 minutes at 4°C, decanted, and cell pellets were resuspended in 3 mL of ACK lysis buffer and incubated for 3 minutes at room temperature. ACK was quenched by adding 10 mL of scRNAseq

buffer (PBS without Ca^{++}/Mg^{++} +0.04% BSA). Samples were centrifuged and washed twice with scRNAseq buffer. 3 samples from each treatment group (vehicle or PNPi) were pooled 1:1:1 at 1000 cells / µL in 1 mL and viability was confirmed to be > 70 % using trypan blue exclusion. Single cell capture and library construction was performed at the UCLA Technology Center for Genomics and Bioinformatics (TCGB) core facility using the 10x Genomics 3'GEX kit. Libraries were sequenced by NextSeq500 High Output at 1x75 read length.

scRNA-seq bioinformatics analysis: Samples were aligned to the mm10 mouse genome using CellRanger (version 4.0.0). The aligned datasets were processed with the Seurat (version 4.0.4) R package in R Studio (version 2021.09.1, R version 4.1.2) (1). For quality control, cells with greater than 20% mitochondrial gene expression and cells with a number of unique molecular identifiers (nUMI) lower than 200 were excluded. Features (genes) not supported by a minimum of 20 cells were excluded. The data from the control and treated samples were integrated using Seurat's IntegrateData function following their default processing pipeline. The integrated data were scaled; any effects of the cell cycle on the single cells' gene expression profile were reduced by regressing out the CellCycle scores of each cell. We regressed the score differences between the S scores and G2M scores of each cell, which were computed using Seurat's CellCycleScoring function. Prior to the scoring, we converted Seurat's built-in human cell cycle genes to their murine counterpart using the biomaRt R package (2). We applied the principal component analysis (PCA) dimensionality reduction. Subsequently, based on the latter's top 30 principal components (PCs), we generated the Uniform Manifold Approximation and Projection (UMAP)-based visualization, nearest-neighbor computation, and cell clustering. Cluster-specific differentially expressed genes (DEGs), computed by Seurat's FindAllMarkers function, were used to identify the cell types of each cluster. Feature plots were generated using the FeaturePlot function (color contrast was increased by using the gene expression-specific color cutoffs for the 10th and 90th quantiles). We obtained treatment-specific DEGs by applying the FindMarkers function to the control and treated cell subsets of each cluster. Gene ontology was analyzed using the built-in Seurat function for EnrichR (DEenrichRPlot) to generate the top 100 DEGs against the MSigDB Hallmark 2020 gene sets.

Flow Cytometry

All flow cytometry data were acquired on a five-laser BD LSRII and analyzed using FlowJo software. For figure data visualization anti-human markers are annotated with "h".

ATO immuno-phenotyping: ATO cells were harvested by adding PBS + 0.5% bovine serum albumin + 2 mM EDTA to each well. The organoid was disaggregated by pipetting and passage through a 50 μm nylon mesh strainer and counted using trypan blue exclusion. CD4/CD8 double-negative or double-positive cells were evaluated within the singlet DAPI$^-$ hCD45$^+$ hCD19$^-$ hCD56$^-$ hCD34$^-$ gated population.

Tissue immuno-phenotyping: Tissues were stored on ice in FACS buffer (PBS+1% FBS) until processing. Spleen, lymph node, and thymic implant tissues were diced and mashed through a 70 μm nylon mesh strainer using a 1 mL syringe plunger in 10 mL of FACS buffer to obtain a single-cell suspension. For red blood cell lysis of spleen samples, suspensions were centrifuged at 450 g for 4 minutes at 4°C, decanted, and cell pellets were resuspended in 3 mL of ACK lysis buffer and incubated for 3 minutes at room temperature. ACK was quenched by adding 10 mL of FACS buffer and samples were passed through a 40 μm nylon mesh filter. $1x10^6$ cells were transferred to a 1.5 mL Eppendorf tube, centrifuged at 450 g for 4 minutes at 4°C, decanted, and resuspended in 100 μL of FACS buffer supplemented with 1:200 diluted fluorochrome-conjugated antibodies and 1:100 diluted Fc-block (anti-mouse CD16/32). After a 20 minute incubation at 4°C, samples were centrifuged and washed twice with 1 mL of FACS buffer before resuspension in 500 μL of FACS buffer + 250 ng/mL DAPI before data acquisition. Antibodies are reported in **Supplemental Table 1**.

Peripheral blood immuno-phenotyping: 200 μL of blood was collected in lithium heparin-coated tubes (BD) using the retro-orbital technique using heparin-coated capillary tubes and stored on ice until processing. Blood was added to 10 mL of ACK buffer and incubated at room temperature for 10 minutes. ACK was quenched by adding 10 mL of FACS buffer and samples were passed through a 40 μm nylon mesh filter. Samples were stained with fluorochrome-conjugated antibodies as described for "tissue immuno-phenotyping".

Cell Trace Violet (CTV) T cell proliferation: Splenocytes were isolated from C57BL/6 mice as described for "tissue immuno-phenotyping". $1x10^7$ splenocytes were resuspended in 1 mL of PBS + 5 μM CTV and incubated in a 37°C water bath for 20 minutes. Stained cells were washed, suspended in 200 μL of RPMI +10%DFBS +30 U/mL IL-2 ± 1 μM PNPi / 5 μM dG with 5 μL of anti-mouse CD3/CD28 DynaBeads. After 72 h of culture, samples were stained with anti-mouse CD3, CD4, and CD8a antibodies and analyzed using flow cytometry. CTV dye dilution was evaluated within CD3/CD4 or CD3/CD8a gated populations.

AnnexinV/PI: Following treatment, cells were washed twice with PBS and incubated with AnnexinV-FITC and propidium iodide diluted in 1x annexin binding buffer per manufacturer's instructions.

Mass Spectrometry

Plasma/Media metabolite extraction, DNA isolation/hydrolysis, and LC-MS/MS-MRM analysis: For stable isotope tracing experiments, cells were collected, washed twice with PBS, and plated at $1x10^6$ cells in 2 mL glucose-free RPMI +10% dialyzed FBS +1 g/L [$^{13}C_6$]glucose ± 5 µM [$^{15}N_5$]dG ± 5 µM [$^{15}N_3$]dC and treated as indicated. Media and DNA analysis were performed on samples obtained from a single well. Intracellular metabolite analysis was performed on samples plated and treated in parallel.

For analysis of media nucleoside abundance and stable isotope composition, a modified version of a previously reported method was applied (3). At experimental endpoints, media was collected in a 1.5 mL microcentrifuge tube, centrifuged at 450xg for 5 minutes at 4°C, and the supernatant was stored at -80°C. For metabolite extraction, 20 µL of supernatant was mixed with 80 µL of 100% MeOH containing stable isotope- labeled nucleoside internal standards (0.5 µM [U-^{15}N/^{13}C]r/dNs). MeOH-extracted samples were incubated at -80°C for 24 hours, centrifuged at 12,000 g for 5 minutes at 4°C and the cleared supernatant was transferred to an HPLC injector vial for analysis.

For analysis of plasma nucleoside and ulodesine levels, blood was collected using a heparin-coated capillary tube by the retro-orbital technique and transferred to a lithium-heparin coated tube on ice. Samples were centrifuged at 450xg for 5 minutes at 4°C and the plasma supernatant was stored at -80°C. For metabolite extraction, 20 µL of plasma was mixed with 80 µL of 100% MeOH containing stable isotope-labeled nucleoside internal standards (0.5 µM [U-^{15}N/^{13}C]r/dNs). MeOH-extracted samples were incubated at -80°C for 24 hours, centrifuged at 12,000 g for 5 minutes at 4°C and the cleared supernatant was transferred to an HPLC injector vial for analysis.

For analysis of stable isotope labeling of nucleosides in DNA, a modified version of a previously reported method was applied (4). Cells were harvested by trypsinization, genomic DNA was extracted using the Quick-gDNA MiniPrep kit, and DNA was hydrolyzed to nucleosides using the DNA Degradase Plus kit following manufacturer-supplied instructions. In the final step of purification, 50 µL of H_2O was used to elute DNA into 1.5 mL microcentrifuge tubes. 50 µL of a DNA nuclease solution (10X buffer/DNA Degradase Plus/H_2O;

2.5/1/1.5) was added to 20 µL of eluted genomic DNA in an HPLC injector vial. Samples were incubated overnight at 37°C before analysis.

LC-MS/MS-MRM of hydrolyzed DNA, plasma, or media was performed as previously described (4). 5 µL of the sample was injected onto a porous graphitic carbon column (Thermo Fisher Scientific Hypercarb, 100 x 2.1 mm, 5 µm particle size) equilibrated in solvent A (0.1% formic acid in MiliQ-purified/LC-Pak treated H_2O) and eluted (700 µL/min) with an increasing concentration of solvent B (0.1% formic acid in acetonitrile) using min/%B/flow rates (µL/min) as follows: 0/2/700, 3/80/700, 4/80/700, 4.5/2/700, 7/2/700. The effluent from the column was directed to an Agilent Jet Stream ion source connected to a triple quadrupole mass spectrometer (Agilent 6460) operating in the multiple reaction monitoring (MRM) mode using previously optimized settings. The peak areas for each target molecule (precursor→fragment ion transitions) at predetermined retention times were recorded using Agilent MassHunter software. Peak areas were normalized to nucleoside internal standard signals. An external standard curve was applied to determine nucleoside or ulodesine concentrations. Experimental and standard samples were processed together to minimize variation.

Intracellular nucleotide extraction and LC-MS/MS-MRM analysis: For intracellular nucleotide analysis, a modified version of a previously reported method was utilized (4). After treatment, cells were collected, centrifuged at 450xg for 4 minutes at 4°C and washed twice with 1 mL of 150 mM NH_4 acetate; and metabolite extraction buffer (80% MeOH in MiliQ-purified/LC-Pak treated H_2O + stable isotope-labeled nucleotide and amino acid internal standards: [^{13}C,^{15}N]amino acids (50 µM) and [U-^{15}N/^{13}C]r/dNTPs (25 µM), [U-^{15}N/^{13}C]r/dNMPs (5 µM), and [U-^{15}N/^{13}C]r/dNs (1 µM)) was added to each sample for a final protein concentration of 0.4 µg / µL. Samples were incubated on ice for 10 minutes, scraped, transferred to 1.5 mL microcentrifuge tubes, vigorously vortexed, and placed on dry ice until sample collection was completed. After incubation at -80°C for 30 minutes, samples were centrifuged at 12,000xg for 15 minutes at 4°C to remove insoluble material. The protein-containing pellet was resuspended in 500 µL of tissue lysis buffer (50 mM ammonium bicarbonate pH 7.2, 0.5% sodium deoxycholate, 12 mM sodium laurel sarcosine), incubated at 4°C for 15 minutes, sonicated and evaluated using the BCA method to determine protein content. In parallel, MeOH-extracted supernatants were transferred to 1.5 mL microcentrifuge tubes and dried in a speed-vac overnight. Dried metabolite pellets were reconstituted in MiliQ-purified/LC-Pak treated H_2O at 5 µL for each 100 µL of metabolite extraction buffer dried (samples were concentrated 20x). Reconstituted samples were vortexed and transferred to HPLC injector vials. 5 µL was injected directly onto a Hypercarb column (100x2.1 mm, 5 µm particle size) equilibrated

in solvent A (5 mM hexylamine and 0.5% diethylamine in MiliQ-purified/LC-Pak treated H_2O, pH adjusted to 10.0 using glacial acetic acid) and eluted (200 µL/min) with an increasing concentration of solvent B (100% acetonitrile) at the following min/%B/flow rates (µL/min): 0/5/200, 15/60/200, 15.5/60/600, 18/5/600, 20.5/5/200, 23/5/200. The effluent from the column was directed to an Agilent Jet Stream ion source connected to a triple quadrupole mass spectrometer (Agilent 6460) operating in the multiple reaction monitoring (MRM) mode using previously optimized settings. The peak areas for each nucleotide (precursor→fragment ion transitions) at predetermined retention times were recorded using Agilent MassHunter software and were normalized to nucleotide internal standards. An external standard curve was applied to determine nucleotide concentrations which were normalized to the protein content of individual samples.

Tumor interstitial fluid analysis: Immediately after isolation, tumors were placed on a 70 µm nylon mesh filter in a 50 mL conical tube and centrifuged at 500xg for 10 minutes at 4°C. Tumor interstitial fluid was collected and processed for LC-MS/MS analysis as derived above for cell culture media samples.

REFERENCES

1. Hao, Y, Hao, S, Andersen-Nissen, E, Mauck, WM, Zheng, S, Butler, A, Lee, MJ, Wilk, AJ, Darby, C, Zager, M, Hoffman, P, Stoeckius, M, Papalexi, E, Mimitou, EP, Jain, J, Srivastava, A, Stuart, T, Fleming, LM, Yeung, B, Rogers, AJ, McElrath, JM, Blish, CA, Gottardo, R, Smibert, P, Satija, R. Integrated analysis of multimodal single-cell data. Cell. 2021;184(13):3573–3587.e29.
2. Durinck, S, Spellman, PT, Birney, E, Huber, W. Mapping identifiers for the integration of genomic datasets with the R/Bioconductor package biomaRt. Nat Protoc. 2009;4(8):1184–1191.
3. Liang, K, Abt, ER, Le, TM, Cho, A, Dann, AM, Cui, J, Li, L, Rashid, K, Creech, AL, Wei, L, Ghukasyan, R, Rosser, EW, Wu, N, Carlucci, G, Czernin, J, Donahue, TR, Radu, CG. STING-driven interferon signaling triggers metabolic alterations in pancreas cancer cells visualized by [^{18}F]FLT PET imaging. Proc Natl Acad Sci U S A. 2021;118(36):e2105390118.
4. Le, TM, Poddar, S, Capri, JR, Abt, ER, Kim, W, Wei, L, Uong, NT, Cheng, CM, Braas, D, Nikanjam, M, Rix, P, Merkurjev, D, Zaretsky, J, Kornblum, HI, Ribas, A, Herschman, HR, Whitelegge, J, Faull, KF, Donahue, TR, Czernin, J, Radu, CG. ATR inhibition facilitates targeting of leukemia dependence on convergent nucleotide biosynthetic pathways. Nat Commun. 2017;8(1):241.

SUPPLEMENTAL TABLE 1

REAGENT or RESOURCE	SOURCE	IDENTIFIER
**Antibodies**		
anti-ACTIN, mouse monoclonal	Cell Signaling Technology	Cat#3700; RRID:AB_2242334
anti-VINCULIN, rabbit monoclonal	Cell Signaling Technology	Cat#13901; RRID:AB_2728768
anti-SAMHD1, mouse monoclonal	ThermoFisher Scientific	Cat# MA5-25354; RRID:AB_2725288
anti-DCK, mouse monoclonal	MilliporeSigma	Cat# MABC188
anti-pCHEK1-S345, rabbit monoclonal	Cell Signaling Technology	Cat#2348; RRID:AB_331212
anti-pH2AX-S139, rabbit monoclonal	Cell Signaling Technology	Cat#9718; RRID:AB_2118009
anti-STAT1, rabbit monoclonal	Cell Signaling Technology	Cat#14994; RRID:AB_2737027
anti-pIKKα/β-S176/180, rabbit monoclonal	Cell Signaling Technology	Cat#2697; RRID:AB_2079382
anti-pNFκB p65-S536, rabbit monoclonal	Cell Signaling Technology	Cat#3033; RRID:AB_331284
anti-pIκBα-S32, rabbit monoclonal	Cell Signaling Technology	Cat#2859; RRID:AB_561111
anti-IKKβ, rabbit monoclonal	Cell Signaling Technology	Cat#11930; RRID:AB_2687618
anti-pNFκB p65, rabbit monoclonal	Cell Signaling Technology	Cat#8242; RRID:AB_10859369
anti-IκBα, 4814	Cell Signaling Technology	Cat#4814; RRID:AB_390781
anti-RABBIT IgG, HRP-LINKED, goat polyclonal	Cell Signaling Technology	Cat#7074; RRID:AB_2099233
anti-MOUSE IgG, HRP-LINKED, goat polyclonal	Cell Signaling Technology	Cat#7076; RRID:AB_330924
anti-human CD45, FITC conjugate,	Beckman Coulter	Cat#IM0782U; RRID:AB_131000
anti-human CD4, BV711 conjugate	BioLegend	Cat#300557; RRID:AB_2564392
anti human, CD8, PE conjugate	BioLegend	Cat#344705; RRID:AB_1953243
anti-mouse CD45, FITC conjugate	BioLegend	Cat#103107; RRID:AB_312972
anti-mouse CD4, BV711 conjugate	BioLegend	Cat#100550; RRID:AB_2562099
anti-mouse CD8, PE conjugate	BioLegend	Cat#100708; RRID:AB_312747
anti-mouse B220, APC-Cy7 conjugate	BioLegend	Cat#103223; RRID:AB_313006
anti-mouse CD11b, FITC conjugate	BioLegend	Cat#101206; RRID:AB_312789
anti-mouse CD11c, APC conjugate	BioLegend	Cat#117309; RRID:AB_313778
anti-mouse F4/80, PE conjugate	BioLegend	Cat#123109; RRID:AB_893498
anti-mouse CD19, BV711 conjugate	BioLegend	Cat#115555; RRID:AB_2565970
anti-mouse GL7, PE conjugate	BioLegend	Cat#144607; RRID:AB_2562925
anti-mouse CD95 (FAS), APC conjugate	BioLegend	Cat#152603; RRID:AB_2632898
anti-mouse TCRβ, FITC conjugate	BioLegend	Cat#109205; RRID:AB_313428
anti-mouse CD62L, PE conjugate	BioLegend	Cat#104407; RRID:AB_313094
anti-mouse PD1, APC-Cy7 conjugate	BioLegend	Cat#135223; RRID:AB_2563522

anti-mouse CXCR5, APC conjugate	BioLegend	Cat#145505; RRID:AB_2561969
TruStain FcX (anti-mouse CD16/32)	BioLegend	Cat#101319; RRID:AB_1574973
**Bacterial and virus strains**		
Stbl3 chemically competent E. Coli	ThermoFisher Scientific	Cat#C737303
**Chemicals, peptides, and recombinant proteins**		
DMEM	Corning	Cat#10-107-CV
RPMI	Corning	Cat#MT10040CV
Glucose-free RPMI	Corning	Cat#MT10043CV
Fetal bovine serum	Omega Scientific	Cat#FB-11
Fetal bovine serum dialyzed	Omega Scientific	Cat#FB-03
B27 supplement	ThermoFisher Scientific	Cat#17504044
Glutamax	ThermoFisher Scientific	Cat#35050061
OptiMEM	ThermoFisher Scientific	Cat#31985-062
Lipofectamine 3000	ThermoFisher Scientific	Cat#L3000001
DOTAP	MilliporeSigma	Cat#11202375001
Ulodesine (PNPi)	WuXi AppTec	N/A
(*R*)-DI-87 (dCKi)	Poddar el al., 2019.	N/A
Human IFNβ	PBL Assay Science	Cat#11415-1
Mouse IFNβ	PBL Assay Science	Cat#11410-2
[$^{13}C_6$]glucose	Sigma-Aldrich	Cat#389374
[$^{15}N_5$]deoxyguanosine	Cambridge Isotope Laboratories	Cat# NLM-3899-CA-5
[$^{15}N_3$]deoxycytidine	Cambridge Isotope Laboratories	Cat#NLM-3897-25
[$^{13}C_9$;$^{15}N_3$]deoxycytidine	Cambridge Isotope Laboratories	Cat# 124603802
deoxyguanosine	MilliporeSigma	Cat#D7145
deoxycytidine	MilliporeSigma	Cat#D3897
guanosine	MilliporeSigma	Cat#G6264
recombinant cytidine deaminase (rCDA)	Abcam	Cat#AB99441
murine MCSF	Peprotech	Cat#315-02
murine FLT3L	Peprotech	Cat#25031L
human FLT3L	Peprotech	Cat#300-19
human IL-7	Peprotech	Cat#200-07
L-ascorbic acid 2-phosphate sesquimagnesium salt hydrate	MilliporeSigma	Cat#A8960
penicillin/streptomycin	Gemini Bio-Products	Cat#400-110

PF-06650833	Selleck Chemicals	Cat#S8531
Takinib	Selleck Chemicals	Cat#S8663
TPCA-1	Selleck Chemicals	Cat#S2824
Dipyridamole	MilliporeSigma	Cat#D9766
NBMPR (S-(4-Nitrobenzyl)-6-thioinosine)	Cayman Chemical	Cat#16403
DAPI (4',6-diamidino-2-phenylindole, dihydrochloride)	ThermoFisher Scientific	Cat#D1306
PNA-FITC Conjugate	MilliporeSigma	Cat#L7381
Dynabeads™ Mouse T-Activator CD3/CD28	ThermoFisher Scientific	Cat#11456D
RIPA protein lysis buffer	Boston BioProducts	Cat#BP-115
Halt protease inhibitor cocktail	ThermoFisher Scientific	Cat#PI78430
Halt phosphatase inhibitor cocktail	ThermoFisher Scientific	Cat#PI78428
Laemmli loading dye	Boston BioProducts	Cat#BP-110R
4-12% Bis-tris protein gels	ThermoFisher Scientific	Cat#NP0336
Nitrocellulose membrane	ThermoFisher Scientific	Cat#88018
Nonfat dry milk	ThermoFisher Scientific	Cat#M-0841
Tris-buffered saline	ThermoFisher Scientific	Cat#50-751-7046
Tween-20	Sigma-Aldrich	Cat#P9416
Supersignal pico	ThermoFisher Scientific	Cat#34580
Supersignal femto	ThermoFisher Scientific	Cat#34095
Autoradiography film	Denville	Cat#E3012
DNA degradase PLUS	Zymo	Cat#E2021
Hard tissue homogenizing vials	Omni International	Cat#19-628
ACK lysis buffer	ThermoFisher Scientific	Cat#A1049201
**Critical commercial assays**		
AnnexinV-FITC apoptosis detection Kit	BD Biosciences	Cat#556547
BCA assay	ThermoFisher Scientific	Cat#23225
Cell Titer Glo	Promega	Cat#G9683
NucleoSpin RNA kit	Takara Bio	Cat#740955.25
MycoPlasma detection Kit	Sigma-Aldrich	Cat#MP0025
EvaGreen qPCR master mix	Lambda Biotech	Cat#MX-S
High-capacity cDNA reverse transcription kit	ThermoFisher Scientific	Cat#4368814

Quick RNA miniprep kit	Zymo	Cat#R1054
Quick genomic DNA miniprep kit	Zymo	Cat#D3021
CD43 (Ly-48) MicroBeads, mouse	Miltenyi	Cat#130-049-801
CD34 MicroBead UltraPure Kit	Miltenyi	Cat#130-100-453
Cell Trace Violet (CTV)	ThermoFisher Scientific	Cat#C34557
**Deposited data**		
RNAseq analysis of BMDM response to TLR7 agonists (Figure 7)	NCBI GEO	GSE203003
**Experimental models: Cell lines**		
Human CCRF-CEM (female) cells	ATCC	Cat#CCL-119; RRID:CVCL_0207
Human JURKAT (male) cells	ATCC	Cat#TIB-152; RRID:CVCL_0367
Human MOLT4 (male) cells	ATCC	Cat#CRL-1582; RRID:CVCL_0013
Human NALM6 (male) cells	ATCC	Cat#CRL-3273; RRID:CVCL_UJ05
Human RS411 (female) cells	ATCC	Cat#CRL-1873; RRID:CVCL_0093
Human SEM (female) cells	DSMZ	Cat#ACC-546; RRID:CVCL_0095
Human MV411 (male) cells	ATCC	Cat#CRL-9591; RRID:CVCL_0064
Human HUT78 (male) cells	ATCC	Cat# TIB-161; RRID:CVCL_0337
Human SUIT2 (male) cells	Accegen	Cat#ABC-TC1175; RRID:CVCL_3172
Human HCC827 (female) cells	ATCC	Cat#CRL-2868; RRID:CVCL_2063
Human M297 cells	This paper	N/A
Human M230 cells	This paper	N/A
Human M257 cells	This paper	N/A
Human M296 cells	This paper	N/A
Human M418 cells	This paper	N/A
MS5-hDLL1 (male) cells	MilliporeSigma	Cat#SCC167; RRID:CVCL_VR88
HS5 (male) cells	ATCC	Cat#CRL-11882; RRID:CVCL_3720
FT293 cells	ThermoFisher Scientific	Cat#R70007; RRID:CVCL_6911
**Experimental models: Organisms/strains**		
Mouse: NOD-*Prkdc*^{em26Cd52}*Il2rg*^{em26Cd22}/NjuCrl (NCG)	Charles River Laboratories	Cat#572; RRID:IMSR_CRL:572
Mouse: NOD.Cg-*Prkdc*^{scid} *Il2rg*^{tm1Wjl}/SzJ (NSG)	Jackson Laboratories	Cat# 005557; RRID:IMSR_JAX:005557
Mouse: C57BL/6J (C57BL/6)	Jackson Laboratories	Cat#000664; RRID:IMSR_JAX:000664
Mouse: BALB/cJ (BALBc)	Jackson Laboratories	Cat#000651; RRID:IMSR_JAX:000651

Mouse: NOD/ShiLtJ (NOD)	Jackson Laboratories	Cat#001976; RRID:IMSR_JAX:001976
Mouse: B6.129S-*Rag2*^{tm1Fwa} *Cd47*^{tm1Fpl} *Il2rg*^{tm1Wjl}/J (TKO)	Jackson Laboratories	Cat#025730; RRID:IMSR_JAX:025730
Mouse: B6.129S1-*Tlr7*^{tm1Flv}/J (TLR7 KO)	Jackson Laboratories	Cat# 008380; RRID:IMSR_JAX:008380
Mouse: B6.129P2(SJL)-*Myd88*$^{tm1.1Defr}$/J (MYD88 KO)	Jackson Laboratories	Cat# 009088; RRID:IMSR_JAX:009088
**Oligonucleotides**		
polyU 21-mer (5'-rUrUrUrUrUrUrUrUrUrUrUrUrUrUrUrUrUrUrUrUrU-3')	IDT	N/A
mouse Il6 RT-PCR primer-forward (5'-CTGCAAGAGACTTCCATCCAG-3')	This paper	N/A
mouse Il6 RT-PCR primer-reverse (5'-AGTGGTATAGACAGGTCTGTT-3')	This paper	N/A
mouse Il10 RT-PCR primer-forward (5'-GGTTGCCAAGCCTTATCGGA-3')	This paper	N/A
mouse Il10 RT-PCR primer-reverse (5'-ACCTGCTCCACTGCCTTGCT-3')	This paper	N/A
mouse Ifnb1 RT-PCR primer-forward (5'-CAGCTCCAAGAAAGGACGAAC-3')	This paper	N/A
mouse Ifnb1 RT-PCR primer-reverse (5'-GGGAGTGTAACTCTTCTGCAT-3')	This paper	N/A
mouse Actin RT-PCR primer-forward (5'-GTATCCTGACCCTGAAGTACC-3')	This paper	N/A
mouse Actin RT-PCR primer-reverse (5'- TGAAGGTCTCAAACATGATCT-3')	This paper	N/A
human SAMHD1-targeting gRNA (5-GTCATCGCAACGGGGACGCT-3')	Liang et al. 2021.	N/A
Human SAMHD1 ORF	NCBI	NM_015474.3
Human CDA ORF	NCBI	NM_001785.2
**Recombinant DNA**		
pMD2G lentiviral packaging plasmid	Addgene	Cat#12259
psPAX2 lentiviral packaging plasmid	Addgene	Cat#12260
LentiCrisprV2 plasmid	Addgene	Cat#52961
pLenti PURO DEST mammalian expression plasmid	Addgene	Cat#17452
pENTR-D/TOPO plasmid	ThermoFisher Scientific	Cat#KP240020
pLenti6/BLOCK-iT™-DEST U6 Gateway® entry vector	ThermoFisher Scientific	Cat#K494300
**Software and algorithms**		
Flowjo software	Tree Star	https://www.flowjo.com
Graphpad Prism software	Graphpad	https://www.graphpad.com/scientific- software/prism/
OsiriX	Pixmeo	https://www.osirix-viewer.com/about/company/
**Other**		
6460 triple quadrupole mass spectrometer	Agilent	N/A

SynergyH1 microplate reader	BioTek	N/A
QuantStudio3 Real-Time PCR system	ThermoFisher Scientific	N/A
G8 microPET/CT scanner	PerkinElmer	N/A
Vevo 2100 ultrasound imaging system	VisualSonics	N/A
